# Pumpkin CmoDREB2A enhances salt tolerance of grafted cucumber through interaction with CmoNAC1 to regulate H_**2**_O_**2**_ and ABA signaling and K^**+**^/Na^**+**^ homeostasis

**DOI:** 10.1093/hr/uhae057

**Published:** 2024-02-28

**Authors:** Yuquan Peng, Lvjun Cui, Ying Wang, Lanxing Wei, Shouyu Geng, Hui Chen, Guoyu Chen, Li Yang, Zhilong Bie

**Affiliations:** National Key Laboratory for Germplasm Innovation & Utilization of Horticultural Crops/College of Horticulture and Forestry Sciences, Huazhong Agricultural University, 430070 Wuhan, China; National Key Laboratory for Germplasm Innovation & Utilization of Horticultural Crops/College of Horticulture and Forestry Sciences, Huazhong Agricultural University, 430070 Wuhan, China; National Key Laboratory for Germplasm Innovation & Utilization of Horticultural Crops/College of Horticulture and Forestry Sciences, Huazhong Agricultural University, 430070 Wuhan, China; National Key Laboratory for Germplasm Innovation & Utilization of Horticultural Crops/College of Horticulture and Forestry Sciences, Huazhong Agricultural University, 430070 Wuhan, China; National Key Laboratory for Germplasm Innovation & Utilization of Horticultural Crops/College of Horticulture and Forestry Sciences, Huazhong Agricultural University, 430070 Wuhan, China; National Key Laboratory for Germplasm Innovation & Utilization of Horticultural Crops/College of Horticulture and Forestry Sciences, Huazhong Agricultural University, 430070 Wuhan, China; National Key Laboratory for Germplasm Innovation & Utilization of Horticultural Crops/College of Horticulture and Forestry Sciences, Huazhong Agricultural University, 430070 Wuhan, China; National Key Laboratory for Germplasm Innovation & Utilization of Horticultural Crops/College of Horticulture and Forestry Sciences, Huazhong Agricultural University, 430070 Wuhan, China; National Key Laboratory for Germplasm Innovation & Utilization of Horticultural Crops/College of Horticulture and Forestry Sciences, Huazhong Agricultural University, 430070 Wuhan, China; Hubei Hongshan Laboratory, Department of Science and Technology of Hubei Province, 430070 Wuhan, China

## Abstract

Pumpkin CmoNAC1 enhances salt tolerance in grafted cucumbers. However, the potential interactions with other proteins that may co-regulate salt tolerance alongside CmoNAC1 have yet to be explored. In this study, we identified pumpkin CmoDREB2A as a pivotal transcription factor that interacts synergistically with CmoNAC1 in the co-regulation of salt tolerance. Both transcription factors were observed to bind to each other’s promoters, forming a positive regulatory loop of their transcription. Knockout of *CmoDREB2A* in the root resulted in reduced salt tolerance in grafted cucumbers, whereas overexpression demonstrated the opposite effect. Multiple assays in our study provided evidence of the protein interaction between CmoDREB2A and CmoNAC1. Exploiting this interaction, CmoDREB2A facilitated the binding of CmoNAC1 to the promoters of *CmoRBOHD1*, *CmoNCED6*, *CmoAKT1;2*, and *CmoHKT1;1*, inducing H_2_O_2_ and ABA synthesis and increasing the K^+^/Na^+^ ratio in grafted cucumbers under salt stress. Additionally, CmoNAC1 also promoted the binding of CmoDREB2A to *CmoHAK5;1*/*CmoHAK5;2* promoters, further contributing to the K^+^/Na^+^ homeostasis. In summary, these findings reveal a crucial mechanism of CmoNAC1 and CmoDREB2A forming a complex enhancing salt tolerance in grafted cucumbers.

## Introduction

Salt stress currently affects approximately 10 × 10^8^ hm^2^ of land worldwide. It is anticipated that by 2050, over 50% of arable land will be impacted by salt stress [1]. As a result, salt stress has emerged as a prominent abiotic stress factor significantly limiting crop productivity [2, 3]. The detrimental mechanisms of salt stress on plants primarily involve:

(i) Ion toxicity: non-selective cation channels facilitate the influx of excessive Na^+^ into plant cells during salt stress, leading to the efflux of intracellular K^+^. Given the critical role of K^+^ in maintaining enzyme activity and cell membrane integrity, the inhibition of K^+^ absorption under salt stress disrupts normal metabolic processes in plant cells [4].

(ii) Oxidative damage: salt stress triggers excessive production of reactive oxygen species (ROS), disrupting redox homeostasis and causing peroxidation of nucleic acids, proteins, and lipids [5].

(iii) Osmotic stress damage: elevated concentrations of Na^+^ increase the osmotic pressure in the soil, hindering the selective absorption capacity of cell membranes and impeding water uptake and transport by plant roots [6].

To mitigate the potential harm caused by salinity stress, plants have developed sophisticated mechanisms [1], including:

(i) Inducing H_2_O_2_ and ABA signaling: under salt stress, plants trigger the production of H_2_O_2_ and ABA to regulate stomatal closure, thus conserving water [6, 7].

(ii) Enhancing the K^+^/Na^+^ ratio: to maintain K^+^/Na^+^ homeostasis under salt stress, plants employ various strategies such as enhancing Na^+^ efflux through upregulating *SOS1* [8], limiting Na^+^ translocation to shoot by upregulating *HKT1* [9], sequestering Na^+^ within vacuoles by upregulating *NHX1* [10], and increasing K^+^ uptake through inducing genes such as *HAK5* and *AKT1* [11].

(iii) Boosting antioxidant capacity: plants upregulate genes encoding antioxidant enzymes, ensuring the synthesis of these enzymes and maintaining ROS homeostasis during salt stress [12].

(iv) Augmenting osmotic regulation capabilities: Plants elevate cellular osmotic pressure by accumulating osmoregulators, thereby preserving osmotic balance under salt stress [13].

DREB2A is classified as a member of the DREB transcription factor family, which can respond to salt stress and regulate salt tolerance in crops such as rice (*Oryza sativa*), soybean (*Glycine max*) and *Arabidopsis thaliana* [14, 15]. DREB transcription factors possess a conserved AP2 domain and exhibit a high degree of specificity in their binding affinity, specifically associating with the DRE *cis*-acting element (A/GCCGAC) [15, 16]. The primary mechanism through which DREB transcription factors regulate plant salt tolerance includes: (i) inducing the biosynthesis of ABA [17] and JA [18]; (ii) enhancing Na^+^ efflux and maintaining K^+^/Na^+^ homeostasis [19]; (iii) enhancing antioxidant enzyme activity and reducing oxidative damage [20]; and (iv) increasing proline content to maintain osmotic balance [20]. Research has shown that DREB transcription factors impact plant salt tolerance by inducing specific gene expression. However, their direct interaction with gene promoters for salt tolerance regulation remains underexplored. Additionally, previous studies have shown that NAC transcription factors have similar functions to DREB transcription factors in regulating plant salt tolerance [21], suggesting that DREB and NAC transcription factors may be involved in co-regulating plant salt tolerance.

In greenhouse vegetable cultivation, the issue of soil secondary salinization is becoming increasingly pronounced due to improper chemical fertilizer application and insufficient rainwater leaching [22]. This issue severely hinders vegetable production in greenhouses. To address this challenge, grafting, as an agronomic technique, has been widely adopted in the cultivation of Cucurbitaceous and Solanaceae vegetables [23–25]. Two notable Cucurbitaceous vegetables are cucumber and pumpkin, with respective annual output values of 3.2 and 1.6 billion dollars (https://www.fao.org/faostat, 2021). While cucumber displays sensitivity to salinity, pumpkin demonstrates robust resistance to salt stress. Grafting cucumber onto pumpkin rootstock has proven effective in improving its salt tolerance. Furthermore, both cucumber and pumpkin possess highly developed vascular tissues, making them suitable subjects for studying grafting signal transduction [26, 27]. The key mechanisms through which grafting improves plant salt tolerance include: (i) upregulation of genes involved in H_2_O_2_ and ABA synthesis in rootstock to facilitate the synthesis and transport of root signals to the shoot [28, 29]; (ii) induction of mobile mRNA movement to the shoot [30]; (iii) restriction of Na^+^ transport to the scion [31] and enhancement of K^+^ absorption [32]; and (iv) increased activity of antioxidant enzymes and elevated proline content in the scion [33, 34]. Our recent study revealed that in grafted cucumbers, CmoNAC1 from the pumpkin rootstock not only interacts with the *CmoRBOHD1* and *CmoNCED6* promoters, promoting H_2_O_2_ and ABA synthesis, but also interacts with the *CmoAKT1;2* and *CmoHKT1;1* promoters to maintain K^+^/Na^+^ homeostasis [21]. Since DREB and NAC transcription factors may have synergistic regulation on the salt tolerance of pumpkin, this study focused on the mechanism of the synergistic regulation of salt tolerance of grafted cucumber by DREB and CmoNAC1 transcription factors.

In this study, we employed a combination of transcriptome analysis, Y2H (yeast two-hybrid) assay, LCI (luciferase complementation imaging), GST Pull-down assay, subcellular localization analysis, LUC (luciferase) assay, Y1H (yeast one-hybrid) assay, and EMSA (electrophoretic mobility shift assay) to elucidate the role of CmoDREB2A as a key transcription factor that interacts with CmoNAC1 in response to salinity. CmoDREB2A was localized in the nucleus and interacted with the promoters of *CmoNAC1*. Conversely, CmoNAC1 was capable of binding to the *CmoDREB2A* promoter. Furthermore, we demonstrated that the interaction between CmoDREB2A and CmoNAC1 promoted the association of CmoNAC1 with the promoters of *CmoRBOHD1*, *CmoNCED6*, *CmoAKT1;2*, and *CmoHKT1;1*, consequently inducing the production of H_2_O_2_ and ABA and increasing the K^+^/Na^+^ ratio. Moreover, our investigation revealed that the interaction between CmoDREB2A and CmoNAC1 also facilitated the binding of CmoDREB2A to the promoters of *CmoHAK5;1* and *CmoHAK5;2*. Overall, this study not only elucidates the mechanism underlying the interaction between CmoDREB2A and CmoNAC1 in regulating salt tolerance in grafted cucumber, but also offers valuable insights for molecular breeding of salt-tolerant pumpkin rootstock.

## Results

### Identification of pumpkin CmoDREB2A as a salt-responsive transcription factor interacting with CmoNAC1

**Figure 1:**
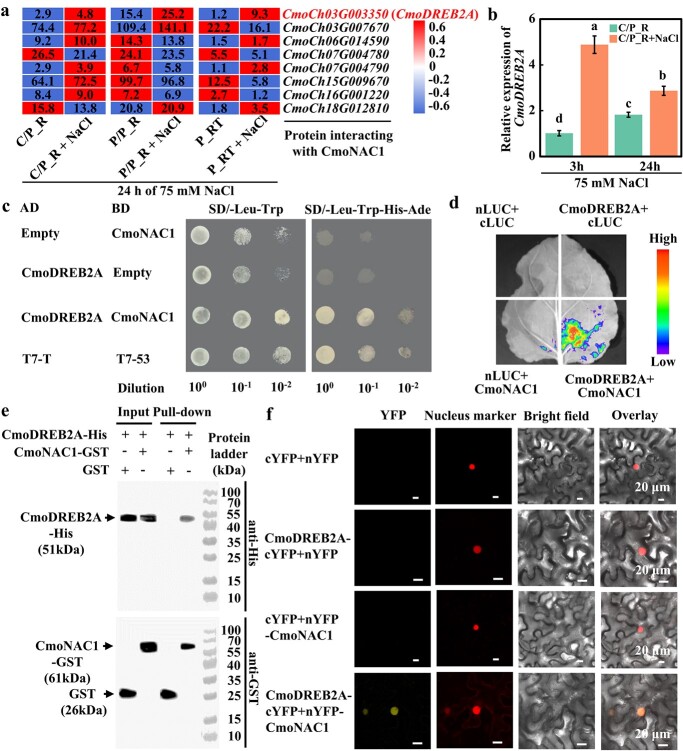
Screening and identification of CmoNAC1 interacting proteins in pumpkin in response to salt stress. (**a**) Expression levels of genes encoding CmoNAC1 interacting proteins found in yeast double sieve library in pumpkin roots treated with 75 mM NaCl for 24 h. C/P_R: root samples of cucumber scion grafted on pumpkin rootstock, P/P_R: root samples of pumpkin scion grafted on pumpkin rootstock, P_RT: pumpkin root tip. (**b**) Expression of *CmoDREB2A* was assessed in pumpkin root samples from cucumber/pumpkin heterografts (C/P_R) treated with 75 mM NaCl for 3 h and 24 h. The different lowercase letters in the figure indicate significant differences between various treatments (*P* < 0.05). (**c**)–(**e**) Verification of CmoDREB2A and CmoNAC1 interaction using yeast two-hybrid assay, LCI, and pull-down assays. (**f**) BiFC analysis of CmoDREB2A and CmoNAC1 interaction.

The aim of this study was to discover proteins that interact with CmoNAC1 in pumpkin. From a pumpkin nuclear protein library, 8 proteins were identified as potential CmoNAC1 interaction partners (Fig. 1a). Among these targets, RNA-seq data showed that only *CmoDREB2A* was up-regulated in response to salt stress in different pumpkin root samples (Fig. 1a). Subsequent qRT-PCR analysis of *CmoDREB2A* exhibited noteworthy upregulation following 3-hour and 24-hour exposure to 75 mM NaCl, in contrast to the control without NaCl (Fig. 1b). These findings imply the crucial involvement of *CmoDREB2A* in the salt stress response of pumpkin rootstocks. To verify the interaction between CmoDREB2A and CmoNAC1, Y2H, LCI, and GST Pull-down experiments were performed. Y2H analysis revealed that yeast co-transformed with AD-Empty vector/BD-CmoNAC1 (negative control), AD-CmoDREB2A/BD-Empty vector (negative control), AD-CmoDREB2A/BD-CmoNAC1, and AD-T7-T/BD-T7–53 (positive control) grew normally on SD/−Leu-Trp medium. However, the negative control yeast failed to grow on SD/−Leu-Trp-His-Ade medium, while the positive control and yeast co-transformed with AD-CmoDREB2A/BD-CmoNAC1 were able to grow (Fig. 1c). This indicates an interaction between CmoDREB2A and CmoNAC1 in yeast cells. LCI analysis demonstrated that tobacco leaves co-transformed with CmoDREB2A/CmoNAC1 exhibited noticeable fluorescence and a significantly increased LUC/REN value compared to those co-transformed with nLUC/cLUC, nLUC/CmoNAC1, or CmoDREB2A/cLUC (Fig. 1d; Fig. S1, see online supplementary material). This further confirms the interaction between CmoDREB2A and CmoNAC1 in tobacco. GST Pull-down experiments showed the presence of bands corresponding to the target protein when CmoDREB2A-His/GST or CmoDREB2A-His/CmoNAC1-GST proteins were added to the respective lanes of Input, as observed through incubation with His or GST antibodies. In the Pull-down lanes, CmoDREB2A-His was pulled down by CmoNAC1-GST, while no interaction was observed with the control GST protein (Fig. 1e). This confirms the *in vitro* interaction between CmoDREB2A and CmoNAC1. BiFC experiments showed that tobacco leaves co-transformed with CmoDREB2A-cYFP/nYFP-CmoNAC1 exhibited noticeable yellow fluorescence compared to those co-transformed with cYFP/nYFP, CmoDREB2A-cYFP/nYFP, or cYFP/nYFP-CmoNAC1. This confirms the *in vivo* interaction between CmoDREB2A and CmoNAC1 (Fig. 1f). Subcellular localization analysis further determined that both CmoDREB2A and CmoNAC1 co-localized with nuclear marker (Fig. S2, see online supplementary material), indicating their presence in the nucleus and supporting the occurrence of their interaction in this cellular compartment.

**Figure 2:**
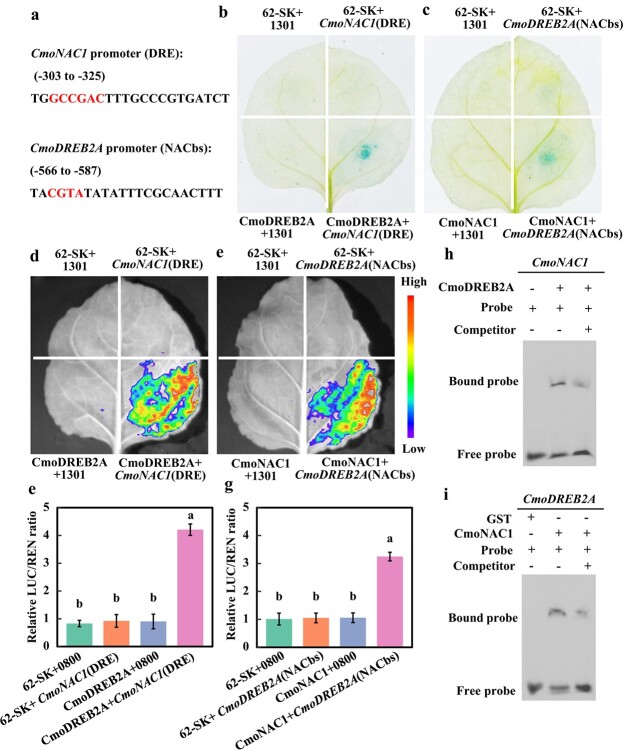
Analysis of the binding of pumpkin CmoDREB2A and CmoNAC1 to each other’s promoter. (**a**) The DRE element of *CmoNAC1* promoter and NACbs of *CmoDREB2A* promoter. (**b**), (**c**) GUS histochemical staining, (**d**)–(**g**) LUC assay, and (**h**), (**i**) EMSA analyses of CmoNAC1 binding to the *CmoDREB2A* promoter, as well as CmoDREB2A binding to the *CmoNAC1* promoter (the probe sequence is the binding site of the transcription factor in (**a**)). The different lowercase letters (**e**, **g**) indicate significant differences (one-way ANOVA, *P* < 0.05).

### Pumpkin CmoDREB2A and CmoNAC1 bind to each other’s promoters

To investigate the potential reciprocal regulation between CmoDREB2A and CmoNAC1, an analysis of their respective promoters revealed the presence of a CmoDREB2A binding element (DRE) on the *CmoNAC1* promoter, as well as a CmoNAC1 binding site (NACbs) on the *CmoDREB2A* promoter (Fig. 2a). The transcriptional activation activity of CmoDREB2A was evaluated. Yeast transformed with BD-CmoDREB2A showed regular growth on SD/-TRP-HIS-Ade medium compared to yeast transformed with BD-Empty vector (Fig. S3, see online supplementary material). This result indicates the presence of transcriptional activation activity in CmoDREB2A. GUS histochemical staining analysis revealed a significant blue coloration in tobacco plants co-transformed with CmoDREB2A and the DRE element of the *CmoNAC1* promoter, in contrast to co-transformations involving 62-SK and 1301, 62-SK and the DRE element of the *CmoNAC1* promoter, or CmoDREB2A and 1301 (Fig. 2b). These findings provide strong evidence for the binding ability of CmoDREB2A to the DRE element of the *CmoNAC1* promoter in tobacco. Similarly, GUS histochemical staining experiments demonstrated that CmoNAC1 binds to the NACbs element of the *CmoDREB2A* promoter (Fig. 2c). LUC analysis demonstrated prominent fluorescence and a significantly increased LUC/RLU ratio in tobacco co-transformed with CmoDREB2A and the DRE element of the *CmoNAC1* promoter, compared to the co-transformations with 62-SK and 0800, 62-SK and the DRE element of the *CmoNAC1* promoter, or CmoDREB2A and 0800 (Fig. 2d and e). These results solidify the capability of CmoDREB2A to bind to the DRE element of the *CmoNAC1* promoter in tobacco. Similarly, CmoNAC1 was found to bind to the NACbs element of the *CmoDREB2A* promoter through LUC experiments (Fig. 2f and g). Furthermore, EMSA analysis indicated that CmoDREB2A protein resulted in a shifted mobility of the 6-FM labeled probe containing DRE element of the *CmoNAC1* promoter, and the subsequent addition of an unlabeled DRE-element probe decreased the intensity of the shifted band (Fig. 2h). This suggests that CmoDREB2A can bind to the DRE element in the CmoNAC1 promoter *in vitro*. Similarly, EMSA analysis confirmed the *in vitro* binding of CmoNAC1 to the NACbs element in the *CmoDREB2A* promoter (Fig. 2i). In conclusion, CmoDREB2A and CmoNAC1 possess the ability to bind to each other’s promoters.

### Effects of knockout and overexpression of *CmoDREB2A* in pumpkin rootstocks on grafted cucumber scions under salt stress

**Figure 3:**
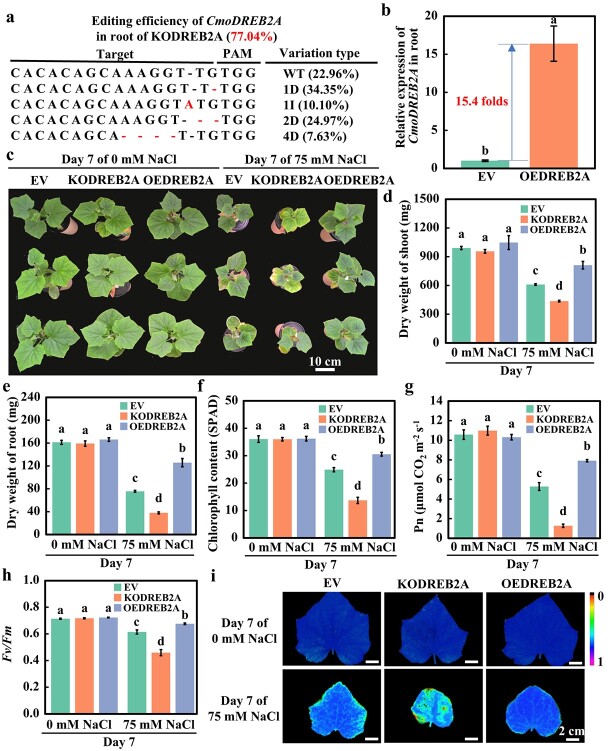
Analysis of phenotypes and photosynthetic indices of cucumbers grafted on *CmoDREB2A*-knockout or overexpression pumpkin rootstock under 75 mM NaCl treatment for 7 days. (**a**) The *CmoDREB2A* gene editing efficiency in *CmoDREB2A*-knockout pumpkin rootstocks (KODREB2A). (**b**) The relative transcript level of *CmoDREB2A* in *CmoDREB2A*-overexpression pumpkin rootstocks (OEDREB2A) compared to the pumpkin roots transformed with empty vector as a control (EV). The different lowercase letters indicate significant differences (*P* < 0.05). (**c**) Phenotypes, (**d**) shoot and (**e**) root dry weights, (**f**) chlorophyll content, (**g**) Pn, and (**h**) value and (**i**) image of *Fv*/*Fm* of cucumbers grafted on the EV, KODREB2A, and OEDREB2A pumpkin rootstocks under 75 mM NaCl treatment for 7 d. Mean ± SE (*n* = 3). Lowercase letters in the figure represent significant differences between various treatments (*P* < 0.05).

To investigate the role of *CmoDREB2A* in salt tolerance of grafted cucumbers, we generated pumpkin rootstocks with knockout (KODREB2A) or overexpression (OEDREB2A) of *CmoDREB2A* using root transformation. The control for this experiment involved pumpkin roots transformed with an empty vector (EV). Hi-TOM analysis revealed an editing efficiency of 77.04% for *CmoDREB2A* in KODREB2A lines (Fig. 3a). qRT-PCR analysis demonstrated a 15.4-fold upregulation of *CmoDREB2A* expression in OEDREB2A compared to EV (Fig. 3b). After subjecting cucumbers grafted onto EV, KODREB2A and OEDREB2A pumpkin rootstocks to 75 mM NaCl treatment for 7 days, we conducted a comprehensive analysis of various parameters. Under normal conditions (0 mM NaCl), the phenotypic characteristics, including shoot and root dry weight, leaf area, root surface area, total root length, and root volume (Fig. 3c–e; Fig. S3a–d, see online supplementary material), photosynthetic indices such as SPAD, Pn, *Fv/Fm*, Gs, Ci, and Tr (Fig. 3f–i; S3e–g, see online supplementary material), and damage indices (REC and MDA contents) (Fig. S4a–d, see online supplementary material) of KODREB2A and OEDREB2A did not exhibit significant changes compared to EV. These results suggest that the growth of grafted cucumber scion remains relatively unaffected by both knockout and overexpression of *CmoDREB2A* in rootstocks under normal condition. However, when treated with 75 mM NaCl for 7 days, the phenotype of KODREB2A deteriorated compared to that of EV (Fig. 3c). The shoot and root dry weight of KODREB2A decreased by 28.4% and 49.8% (Fig. 3d and e), compared to those of EV, respectively. Along with significant reductions in leaf area, root surface area, total root length, and root volume (Fig. S4a–d, see online supplementary material). There was also a notable decline in photosynthetic capacity, with SPAD and Pn decreasing by 45.0% and 75.9% (Fig. 3f and g), respectively, and significant decreases observed in Fv/Fm, Gs, Ci, and Tr (Fig. 3h; Fig. S3e–g, see online supplementary material). Furthermore, there was a considerable increase in MDA content and REC in both leaves and roots (Fig. S5a–d, see online supplementary material). On the other hand, OEDREB2A exhibited a significantly improved phenotype under salt stress compared to EV (Fig. 3c). The shoot and root dry weight increased by 33.0% and 66.4% (Fig. 3d and e), respectively, and there were significant increases in leaf area, root surface area, total root length, and root volume (Fig. S4a–d, see online supplementary material). Additionally, photosynthetic capacity was enhanced, with SPAD and net photosynthetic rate (Pn) increasing by 22.6% and 50.0% (Fig. 3f and g), respectively, and significant increases in maximum photochemical efficiency (*Fv/Fm*), intercellular CO_2_ concentration (Ci), stomatal conductance (Gs), and transpiration rate (Tr) (Fig. 3h; Fig. S3e–g, see online supplementary material). Damage levels were also reduced, as evidenced by significantly reduced MDA content and REC in both leaves and roots (Fig. S5a–d). Altogether, these results demonstrate that *CmoDREB2A* positively regulates salt tolerance in grafted cucumbers*.*

### The interaction between CmoDREB2A and CmoNAC1 facilitates the production of H_2_O_2_ and ABA in grafted cucumber under salt stress

**Figure 4:**
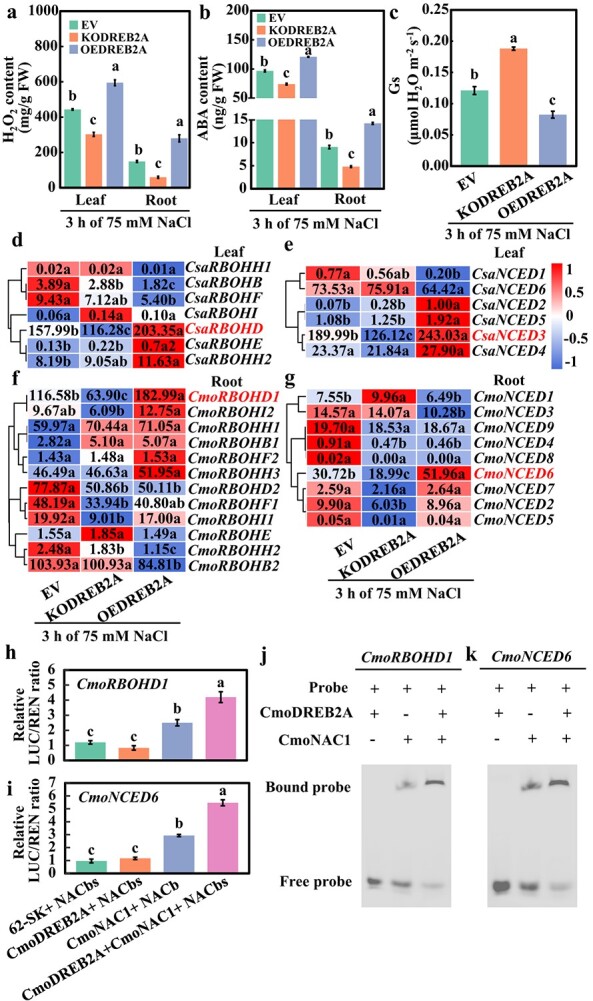
Analysis of H_2_O_2_ and ABA signaling in cucumbers grafted on *CmoDREB2A*-knockout or overexpression pumpkin rootstock after a 3-hour treatment with 75 mM NaCl. (**a**) H_2_O_2_ and (**b**) ABA contents, (**c**) stomatal conductance of leaves, and (**d**)–(**g**) expression levels of genes involved in H_2_O_2_ (*RBOHs*) and ABA (*NCEDs*) synthesis in cucumbers grafted on *CmoDREB2A*-knockout (KODREB2A) or overexpression (OEDREB2A) pumpkin rootstocks following the 3-hour treatment with 75 mM NaCl. Pumpkin roots transformed with empty vector was used as a control (EV). (**h**), (**i**) LUC and (**j**), (**k**) EMSA assay was used to analyse the influence of CmoDREB2A on the binding of CmoNAC1 to the promoters of *CmoRBOHD1* and *CmoNCED6*. Different lowercase letters in the figure indicate significant differences between various treatments (*P* < 0.05).

To investigate the impact of CmoDREB2A on H_2_O_2_ and ABA synthesis, as well as stomatal conductance in leaves under salinity stress, the H_2_O_2_ content, ABA level, and stomatal conductance were quantified in cucumbers grafted on EV, KODREB2A, and OEDREB2A pumpkin rootstocks. Results showed that following a 3-hour treatment with 75 mM NaCl, the H_2_O_2_ content in both leaves and roots of KODREB2A declined by 31.8% and 60.0%, respectively, compared to the control (EV) (Fig. 4a). Similarly, the ABA content in leaves and roots of KODREB2A exhibited a reduction of 23.3% and 47.0%, respectively, while the stomatal conductance of leaves increased by 55.4% (Fig. 4b and c). Conversely, the H_2_O_2_ content in leaves and roots of OEDREB2A increased by 34.1% and 87.6% (Fig. 4a), ABA content in leaves and roots increased by 25.1% and 56.7% (Fig. 4b), respectively, and stomatal conductance of leaves decreased by 32.0% (Fig. 4c). To further elucidate the impact of *CmoDREB2A* on the expression of genes related to H_2_O_2_ and ABA synthesis, RNA-seq analysis was performed. PCA analysis of RNA-seq data revealed that the three replicates of each treatment fell within a 95% confidence ellipse, indicating excellent sample repeatability (Fig. S6a and b, see online supplementary material). We verified the expression of 10 genes through qRT-PCR, and the high correlation of 94% between them indicates the accuracy and reliability of the transcriptome data (Fig. S6c and d, see online supplementary material). To investigate how CmoDREB2A regulates salt tolerance in grafted cucumbers, differentially expressed genes (DEGs) were analysed. Compared to EV, cucumber grafted on KODREB2A and OEDREB2A pumpkin rootstocks displayed 255 and 1450 up-regulated genes and 246 and 1169 down-regulated genes in leaves (Fig. S7a, see online supplementary material), 129 and 356 up-regulated genes and 955 and 324 down-regulated genes in roots (Fig. S7b, see online supplementary material), respectively. A total of 329 DEGs were shared in the leaves of KODREB2A and OEDREB2A, while 277 DEGs were shared in the roots (Fig. S7c and d, see online supplementary material). GO enrichment analysis revealed that the 329 DEGs in leaves were enriched in molecular functions such as cation binding and oxidoreductase activity (Fig. S7e, see online supplementary material). Additionally, the 277 DEGs in roots were enriched in molecular functions such as transporter activity and oxidoreductase activity (Fig. S6f, see online supplementary material). This suggests that CmoDREB2A plays a role in ion transport and oxidation regulation in grafted cucumbers under salt stress.

**Figure 5:**
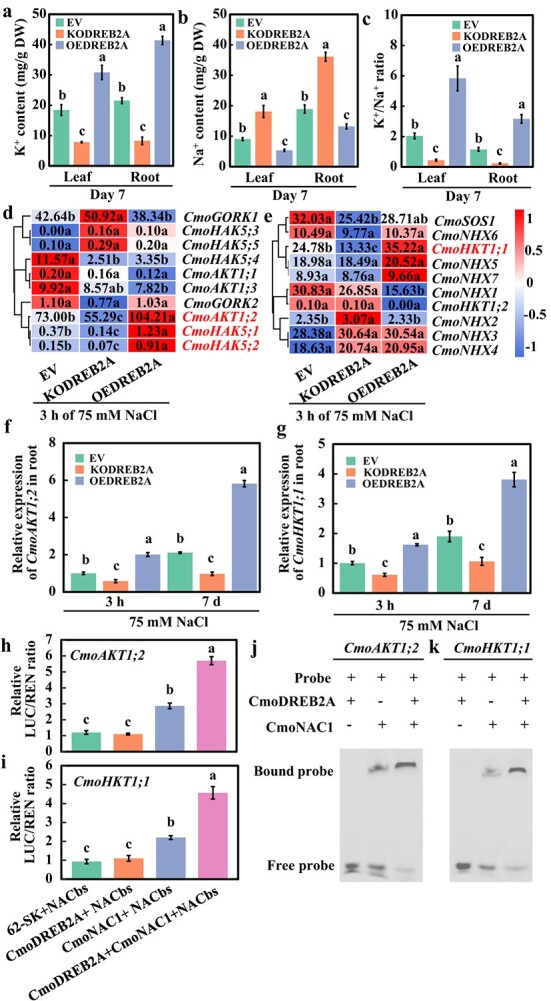
K^+^/Na^+^ homeostasis in cucumbers grafted on *CmoDREB2A*-knockout or overexpression pumpkin rootstock under salt stress conditions. (**a**) K^+^ and (**b**) Na^+^ contents, (**c**) K^+^/Na^+^ ratio, and the expression levels of genes associated with (**d**) K^+^ and (**e**) Na^+^ transport in in cucumbers grafted on *CmoDREB2A*-knockout (KODREB2A) or overexpression (OEDREB2A) pumpkin rootstocks treated with 75 mM NaCl for 7 d. Pumpkin roots transformed with empty vector was used as a control (EV). (**f**), (**g**) qRT-PCR analysis of the expression levels of *CmoAKT1;2* and *CmoHKT1;1* in EV, KODREB2A, and OEDREB2A rootstocks treated with 75 mM NaCl for 3 h. (**h**), (**i**) LUC and (**j**), **(k**) EMSA analysis of the interaction between CmoDREB2A and CmoNAC1 on the binding of CmoNAC1 to the promoters of *CmoAKT1;2* and *CmoHKT1;1*. The lowercase letters in the figure represent significant differences among the different treatments (*P < 0.05*).

Subsequently, we examined the expression levels of *RBOHs* and *NCEDs* regulating H_2_O_2_ and ABA synthesis. It was observed that, when compared to EV, only *CsaRBOHD* and *CsaNCED3* in the leaves of KODREB2A and OEDREB2A exhibited significant alterations, whereas only *CmoRBOHD1* and *CmoNCED6* demonstrated notable changes in the roots (Fig. 4d–g). Their expression levels were validated through qRT-PCR, revealing that, in comparison to EV, *CsRBOHD* and *CsNCED3* decreased by 46.4% and 54.3%, respectively, in KODREB2A leaves, while they increased by 63.1% and 64.8%, respectively, in OEDREB2A leaves (Fig. S8a, see online supplementary material). In the roots, KODREB2A led to a decrease of 56.7% and 65.2% in *CmoRBOHD1* and *CmoNCED6*, respectively; conversely, OEDREB2A resulted in an increase of 95.1% and 111.1% in *CmoRBOHD1* and *CmoNCED6*, respectively (Fig. S8b, see online supplementary material). To elucidate the regulatory effects of CmoDREB2A and CmoNAC1 interaction on the expression of *CmoRBOHD1* and *CmoNCED6*, LUC experiments were conducted in tobacco. Compared to co-transformation of CmoNAC1 and NACbs of the *CmoRBOHD1* promoter, the co-transformation of CmoNAC1, CmoDREB2A, and NACbs of the *CmoRBOHD1* promoter resulted in a significant increase of 67.0% in the LUC/RLU ratio (Fig. 4h). Similarly, when co-transformed CmoNAC1, CmoDREB2A, and NACbs of the *CmoNCED6* promoter, the LUC/RLU ratio exhibited a substantial increase of 87.0% compared to the co-transformed CmoNAC1 and NACbs of the *CmoNCED6* promoter (Fig. 4i). Consistent with the findings of LUC experiments, EMSA *in vitro* also affirmed the interaction between CmoDREB2A and CmoNAC1 facilitates the binding of CmoNAC1 to the promoters of *CmoRBOHD1* and *CmoNCED6* (Fig. 4j and k). Overall, this indicates that the interaction between CmoDREB2A and CmoNAC1 facilitates the binding of CmoNAC1 to the promoters of two key genes in H_2_O_2_ and ABA generation in grafted cucumbers in response to salinity.

### The interaction between CmoDREB2A and CmoNAC1 facilitates the K^+^/Na^+^ homeostasis in grafted cucumber under salt stress

To investigate the role of CmoDREB2A in regulating K^+^/Na^+^ homeostasis in grafted cucumbers under salt stress, we analysed K^+^/Na^+^ contents in cucumbers grafted on EV, KODREB2A, and OEDREB2A pumpkin rootstocks. Results showed that following exposure to 75 mM NaCl for 7 days, leaves and roots of KODREB2A exhibited a decline of 57.6% and 61.5% in K^+^ content, and an elevation of 100.3% and 91.0% in Na^+^ content, leading to a reduction of 78.3% and 80.0% in K^+^/Na^+^ ratio, respectively, compared to EV (Fig. 5a–c). Conversely, leaves and roots of OEDREB2A demonstrated an increase of 67.2% and 92.1% in K^+^ content, and a decrease of 40.5% and 30.2% in Na^+^ content, resulting in an upsurge of 185.7% and 173.8% in K^+^/Na^+^ ratio, respectively, compared to EV (Fig. 5a–c). Expression analysis of genes associated with K^+^/Na^+^ transport revealed significant changes in K^+^ transporter genes *CmoAKT1;2*, *CmoHAK5;1*, and *CmoHAK5;2*, and Na^+^ transporter gene *CmoHKT1;1* when exposed to 75 mM NaCl for 3 h in both KODREB2A and OEDREB2A rootstock compared to EV (Fig. 5d and e). qRT-PCR verification demonstrated that *CmoAKT1;2* was downregulated by 53.9% and 41.9% (Fig. 5f), *CmoHKT1;1* was downregulated by 44.0% and 39.0% (Fig. 5g), *CmoHAK5;1* was downregulated by 47.0% and 68.1% (Fig. S8c, see online supplementary material), and *CmoHAK5;2* was downregulated by 44.5% and 72.3% (Fig. S8d, see online supplementary material) in KODREB2A roots following exposure to 75 mM NaCl for 3 h and 7 d compared to EV, respectively. Meanwhile, in OEDREB2A roots, *CmoAKT1;2* expression elevated by 176.0% and 100.3% (Fig. 5f), *CmoHKT1;1* expression increased by 100.7% and 61.5% (Fig. 5g), *CmoHAK5;1* expression upregulated by 163.1% and 74.7% (Fig. S7c, see online supplementary material), and *CmoHAK5;2* expression upregulated by 125.6% and 66.5% (Fig. S7d, see online supplementary material) following 75 mM NaCl treatment for 3 h and 7 d compared to EV, respectively. Given the ability of CmoNAC1 to bind to the NACbs regions within the promoters of *CmoAKT1;2* and *CmoHKT1;1*, we examined how the interaction between CmoDREB2A and CmoNAC1 affects the regulation of *CmoAKT1;2* and *CmoHKT1;1* by CmoNAC1 using LUC assay. Notably, our results demonstrated that co-transformation of CmoDREB2A, CmoNAC1, and NACbs of the *CmoAKT1;2* promoter resulted in a significant elevation of LUC/RLU ratio by 99.0% relative to co-transformation of CmoNAC1 and NACbs of the *CmoAKT1;2* promoter (Fig. 5h). In addition, co-transformation of CmoDREB2A, CmoNAC1, and NACbs of the *CmoHKT1;1* promoter led to an increase of LUC/RLU ratio by 106.6% compared to co-transformation of CmoNAC1 and NACbs of the *CmoHKT1;1* promoter (Fig. 5i). And EMSA *in vitro* also affirmed the interaction between CmoDREB2A and CmoNAC1 facilitates the binding of CmoNAC1 to the promoters of *CmoAKT1;2* and *CmoHKT1;1* (Fig. 5j and k). These observations suggest that the interaction between CmoDREB2A and CmoNAC1 facilitate the binding of CmoNAC1 to the *CmoAKT1;2*/*CmoHKT1;1* promoters, ultimately promoting K^+^/Na^+^ homeostasis in grafted cucumber plants challenged by salt stress.

### Pumpkin CmoDREB2A regulates K^+^ absorption under salinity stress

**Figure 6:**
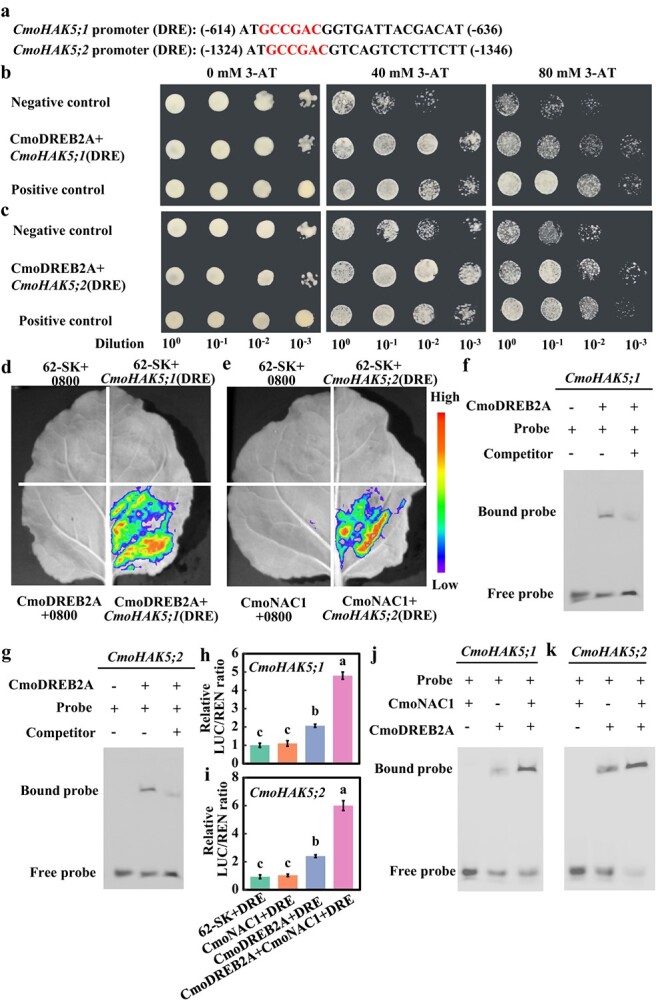
Analysis of CmoDREB2A binding to the *CmoHAK5;1* and *CmoHAK5;2* promoters. (**a**) DRE element within the *CmoHAK5;1* and *CmoHAK5;2* promoter. (**b**), (**c**) Yeast one-hybrid, (**d**), (**e**) LUC assay, and (**f**), (**g**) EMSA analysis of the binding between CmoDREB2A and the *CmoHAK5;1* and *CmoHAK5;2* promoters (The probe sequence is the binding site of the CmoDREB2A in (**a**)). (**h**), (**i**) LUC and (**j**), (**k**) EMSA assay was used to analyse the impact of CmoNAC1 on the binding of CmoDREB2A to the *CmoHAK5;1* and *CmoHAK5;2* promoters. The lowercase letters in the figure denote significant differences observed among the various treatments (*P < 0.05*).

To elucidate the mechanism underlying the CmoDREB2A-regulated K^+^ absorption, an analysis of the promoters for both *CmoHAK5;1* and *CmoHAK5;2* genes revealed the presence of DRE elements (Fig. 6a). Y1H analysis demonstrated that co-transformation of CmoDREB2A with the DRE element from the *CmoHAK5;1* and *CmoHAK5;2* promoters enabled yeast growth at a dilution of 10^−3^ in the presence of 80 mM 3-AT, unlike the negative control (Fig. 6b and c). These results confirm the binding ability of CmoDREB2A to the DRE elements within the promoters of *CmoHAK5;1* and *CmoHAK5;2*. Consistent with these findings, LUC assays conducted in tobacco and *in vitro* EMSA also affirmed the interaction between DREB2A and the DRE elements within the *CmoHAK5;1* and *CmoHAK5;2* promoters (Fig. 6d–g). Subsequently, LUC analysis was employed to investigate the cooperative effect of CmoDREB2A and CmoNAC1 on the regulation of *CmoHAK5;1* and *CmoHAK5;2*. Notably, co-expression of CmoNAC1 with CmoDREB2A and the DRE elements of the *CmoHAK5;1/CmoHAK5;2* promoters resulted in a significant increase in the LUC/RLU ratio, compared to CmoDREB2A and DRE elements alone (Fig. 6h and i). Similarity, EMSA *in vitro* also affirmed the interaction between CmoDREB2A and CmoNAC1 facilitates the binding of CmoDREB2A to the promoters of *CmoHAK5;1* and *CmoHAK5;2* (Fig. 6j and k) These results strongly indicate that the interaction between CmoNAC1 and CmoDREB2A enhances the binding of CmoDREB2A to the *CmoHAK5;1* and *CmoHAK5;2* promoters, ultimately facilitating K^+^ absorption in grafted cucumbers plants in response to salinity conditions.

## Discussion

### Pumpkin CmoDREB2A is a key transcription factor interacting with CmoNAC1 and positively regulates salt tolerance of grafted cucumbers

Under conditions of salt stress and osmotic stress, the proteins that interact with NAC transcription factors and their associated regulatory mechanisms exhibit variations among different species [15]. For instance, in sweet potato, IbNAC3 has the capability to interact with ANAC072 and NAP, forming a protein complex that synergistically activates the expression of the E3 ubiquitin ligase MREL57, thus enhancing salt tolerance [35]. Similarly, in tomato, the NAC transcription factor SlVOZ1 interacts with protein kinase SlOST1, which phosphorylates its serine residue at the 67th position, leading to a positive regulation of osmotic stress tolerance [36]. Moreover, in rice, the protein kinases GSK2 and SAPK8 can interact with OsNAC016, promoting its phosphorylation and subsequently reducing its stability while enhancing osmotic stress tolerance [37, 38]. In Arabidopsis, the PwNAC11 protein of spruce interacts with DREB2A and ABF3 from Arabidopsis, thereby participating in the regulation of osmotic stress tolerance [39]. Simultaneously, distinct DREB transcription factors govern plant salt tolerance across various species. In tomato, enhancing its salt tolerance entails overexpressing *SlDREB2* [17]. Similarly, in soybean and tobacco, elevating salt tolerance involves overexpressing *GmDREB6* from soybean [40]. *A. thaliana*, on the other hand, exhibits improved salt tolerance through overexpression of *TaDREB3* from wheat [41], *DREB2A* from *Pennisetum glaucum* [42], and *ScDREB5* from *Syntrichia caninervis* [18]. In cultivated soybeans, the reduction in salt tolerance is attributed to the loss of the *DREB3b*^39Del^ allele [43].

This study identified CmoDREB2A as a key transcription factor responding to salt stress by interacting with CmoNAC1 through yeast double-hybrid sieve library and transcriptome analysis (Fig. 1a and b). The interaction was verified by dual luciferase complementary experiments and GST Pull-down (Fig. 1c–e). Interestingly, unlike the interaction partners of NAC transcription factors in sweet potato [35], tomato [36], and rice [37, 38], CmoDREB2A was found to interact with CmoNAC1 in pumpkin, possibly due to the presence of different proteins and NAC transcription factors across species, as well as the screen limitation of the current library. Further screening is needed to identify more proteins interacting with CmoNAC1, such as protein kinase and ubiquitin ligase. Furthermore, the interaction between CmoNAC1 and CmoDREB2A of pumpkin was found to resemble that between PwNAC11 of spruce and DREB2A of Arabidopsis [39]. However, CmoNAC1 and PwNAC11 display a low protein sequence similarity of 44.65%, indicating potential differences in their interactions. In this study, three methods were employed to verify the interaction, leading to more robust and reliable results compared to the sole use of yeast two-hybrid assay in the case of PwNAC11 and AtDREB2A [39]. Furthermore, our study unveiled that the interaction between CmoDREB2A and CmoNAC1 takes place within the nucleus (Fig. 1f). Notably, as shown in Fig. 2a–i, both CmoDREB2A and CmoNAC1 were found to bind to each other’s promoters, implying a potential mechanism wherein they mutually modulate salt tolerance by regulating the transcript level of each other. This novel finding has not been reported in previous studies on regulation of plant salt tolerance by NAC and DREB2A transcription factors. In this study, it was noted that CmoDREB2A positively contributed to the salt tolerance of grafted cucumber (Fig. 3a–i), mirroring the findings in tomato [17], tobacco [40], Arabidopsis [18, 41, 42], and soybean [43]. Interestingly, it has been demonstrated that various DREB transcription factors are involved in the salt tolerance in tomato [17], soybean [40, 43], and wheat [41]. These findings suggest potential differences in the regulatory mechanisms across different species and highlighting the importance of exploring diverse models. It is worth noting that while previous studies often relied on the overexpression of DREB transcription factors to validate their functionality [17, 18, 40–42], this study employed gene editing techniques to further confirm the function of *CmoDREB2A*, enhancing the reliability of the results.

### The interaction between CmoDREB2A and CmoNAC1 promoted the binding of CmoNAC1 to its target gene promoters

H_2_O_2_ and ABA signals are crucial early indicators of plant responses to salt and osmotic stresses, and their production is regulated by different DREB transcription factors across different plant species [15]. In tomatoes, *SlDREB2* promotes ABA signaling by downregulating the expression of the abscisic acid 8′-hydroxylase 1-like (*ABAhydroxy*) [17]. Likewise, in tobacco, sugarcane *ScDREB2B-1* regulates H_2_O_2_ and ABA signal production by upregulating *NbRbohB* and *NbNCED* to combat osmotic stress [44]. These signals also play a vital role in vegetable grafting, where overexpression of *SlNCED1* in grafted tomato rootstocks promotes ABA generation and translocation to scion leaves, thus increasing salt tolerance [29]. Similarly, in grafted peppers, salt-tolerant rootstock enhances H_2_O_2_ level in scion leaves to activate antioxidant capacity, as well as ABA production to promote leaf stomata closure, thereby improving its salt tolerance [27]. Cucumber grafts also benefit from pumpkin rootstock *CmoNAC1*, which binds to *CmoRBOHD1* and *CmoNCED6* promoters, resulting in increased production and transport of H_2_O_2_ and ABA to cucumber scions, promoting leaf stomatal closure and salt stress resistance [34, 45, 46].

In this study, it was observed that *CmoDREB2A* in pumpkin plays a regulatory role in the production of H_2_O_2_ and ABA signaling by upregulating *CmoRBOHD1* and *CmoNCED6* (Fig. 4a–g). This mechanism is similar to the findings in tobacco [44] but differs from the regulatory mechanisms identified in tomato [17], suggesting potential species-specific differences. Additionally, our study revealed that CmoDREB2A interacts with CmoNAC1, facilitating the binding of CmoNAC1 to the promoter regions of its target genes (Fig. 4h and i). This aspect of the investigation provides a comprehensive understanding beyond previous studies conducted in tobacco [44] and tomato [17]. Furthermore, the study uncovered that CmoDREB2A interacts with CmoNAC1 in promoting the production of H_2_O_2_ and ABA signals in pumpkin rootstock, offering deeper insights compared to the studies conducted on grafted tomatoes [29] and grafted peppers [27]. Concurrently, the study advances the current understanding of CmoNAC1’s role in regulating H_2_O_2_ and ABA signaling of grafted cucumbers [34]. Notably, the study also identified CmoDREB2A’s ability to bind to the promoter region of *CmoNAC1* (Fig. 2a, b, d, e, and h), suggesting its capacity to modulate H_2_O_2_ and ABA signal production by regulating *CmoNAC1* expression.

### The CmoDREB2A-CmoNAC1 complex enhances the K^+^/Na^+^ ratio in grafted cucumbers under salinity stress

DREB transcription factors have the capacity to enhance plant resistance to salinity stress by modulating K^+^/Na^+^ ratios in various species. In tomato, SlDREB2 is required in increasing K^+^ uptake and decreasing Na^+^ absorption, thereby improving salt tolerance [17]. Similarly, PvDREB1C induces *NtNHX4* expression and elevates the K^+^/Na^+^ ratio in tobacco [47]. Overexpression of sorghum *DREB2* in maize also enhances K^+^/Na^+^ ratios [48]. In *A. thaliana*, *Salix matsudana* SmDREBA1–4 binds to the *AtSOS1* promoter and enhances K^+^/Na^+^ ratios [19], while *S. caninervis* ScDREB5 promotes Na^+^ exodulation by inducing *SOS1*/*SOS2*/*SOS3* expression [18]. Grafting also increases the K^+^/Na^+^ ratio and improves salt tolerance in vegetable crops. For instance, grafted cucumbers exhibit upregulated *HAK5* expression in both pumpkin rootstocks and cucumber scions, leading to increased K^+^ uptake [31, 49]. Additionally, pumpkin rootstock enhances the K^+^/Na^+^ ratio by CmoNAC1 binding to *CmoHKT1;1* and *CmoAKT1;2* promoters [21].

In this study, the regulatory role of *CmoDREB2A* in salt tolerance was investigated, focusing on its impact on K^+^ absorption, Na^+^ absorption, and the K^+^/Na^+^ ratio. Similar to findings in tomato [17], tobacco [47], maize [48], and Arabidopsis [18, 19], *CmoDREB2A* was found to enhance salt tolerance by increasing K^+^ absorption and decreasing Na^+^ absorption, resulting in an improved K^+^/Na^+^ ratio (Fig. 5a–c). However, unlike the regulatory mechanisms observed in tobacco [47] and Arabidopsis [18, 19], *CmoDREB2A* was found to promote Na^+^ transport through *CmoHKT1;1* and K^+^ absorption through *CmoAKT1;2*, *CmoHAK5;1*, and *CmoHAK5;2* (Fig. 5d–g). This discrepancy may be attributed to variations in DREB transcription factors and species-specific factors. Additionally, the study corroborated previous findings that pumpkin rootstocks can enhance the K^+^/Na^+^ ratio through the regulation of *CmoHKT1;1* [31], *CmoHAK5;1*, and *CmoHAK5;2* [49]. Furthermore, while previous studies identified the direct binding of CmoNAC1 to the promoters of *CmoHKT1;1* and *CmoAKT1;2* [34], our investigation revealed that CmoDREB2A facilitates the interaction between CmoNAC1 and the promoters of *CmoHKT1;1* and *CmoAKT1;2* (Fig. 5h and i). This novel mechanism further enriches our understanding of how CmoNAC1 regulates salt tolerance in grafted cucumbers. Notably, our findings revealed that CmoDREB2A plays a crucial role in facilitating K^+^ absorption by modulating the expression of *CmoHAK5;1* and *CmoHAK5;2* (Fig. 6a–g). Additionally, we made a noteworthy discovery wherein the interaction between CmoNAC1 and CmoDREB2A enhances the binding ability of CmoDREB2A to the promoters of *CmoHAK5;1* and *CmoHAK5;2* (Fig. 6h and i), marking a significant departure from previous research [34]. These findings highlight the distinct regulatory mechanisms employed by different transcription factors in modulating K^+^/Na^+^ transporters during salt stress in pumpkin rootstocks.

**Figure 7:**
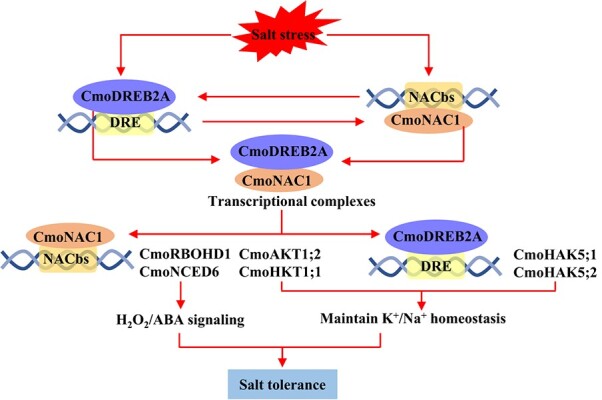
The underlying mechanism of the interaction between CmoDREB2A and CmoNAC1 in regulating salt tolerance of grafted cucumbers. Both CmoDREB2A and CmoNAC1 are capable of binding to each other’s promoters. The transcriptional complexes formed by CmoDREB2A and CmoNAC1 not only enhance the binding affinity of CmoNAC1 to the *CmoRBOHD1* and *CmoNCED6* promoters, leading to increased production of H_2_O_2_ and ABA, but also facilitate the binding of CmoNAC1 to the *CmoAKT1;2* and *CmoHKT1;1* promoter and the binding of CmoDREB2A to the *CmoHAK5;1* and *CmoHAK5;2* promoter, thereby promoting K^+^/Na^+^ homeostasis.

In conclusion, the pivotal role of CmoDREB2A in regulating K^+^/Na^+^ homeostasis in grafted cucumbers is evident under salt stress (Fig. 7). It not only binds to the promoter of *CmoNAC1* but also interacts with CmoNAC1 to facilitate the binding of CmoNAC1 to the promoters of its target genes, including *CmoRBOHD1* and *CmoNCED6* involved in the H_2_O_2_ and ABA synthesis, respectively, and *CmoAKT1;2* and *CmoHKT1;1* regulating K^+^/Na^+^ homeostasis. Moreover, CmoNAC1 interacts with CmoDREB2A and promotes the binding of CmoDREB2A to the promoters of *CmoHAK5;1*/*CmoHAK5;2*, regulating K^+^ uptake under salt stress.

## Materials and methods

### Yeast two-hybrid screening

To discover proteins that engage with CmoNAC1, we conducted a yeast two-hybrid screening using a nuclear library from pumpkin. Following the protocol described in a previous study [50], the bait vector pGBKT7 integrating the coding sequence of CmoNAC1 was utilized, while the prey vector pGADT7 harboring the pumpkin nuclear library was used. The transformed vectors were introduced into Y2HGold yeast and subjected to yeast two-hybrid screening following the manufacturer’s instructions (Clontech Company). To verify the interaction between CmoNAC1 and CmoDREB2A, Y2H assay was performed according to a previously established method [9]. Specifically, *CmoNAC1* and *CmoDREB2A* were cloned onto pGADT7 and pGBKT7 vectors using BamH1 enzyme, respectively, which were then transformed into Y2H Gold competent cells. Positive clones were selected from SD/−Leu/−Trp screening medium and subsequently inoculated onto SD/−Leu/−Trp/-His/−Ade solid medium to detect the interaction and self-activation activity of CmoNAC1 and CmoDREB2A. The primers for cloning are listed in Table S1 (see online supplementary material).

### Transcriptome data

The transcriptome data for pumpkin roots exposed to 75 mM NaCl for 24 hours can be accessed from the following bioproject in NCBI website: PRJNA952931 (transcriptome data for grafted cucumber and self-grafted pumpkin roots) [32], PRJNA437579 (transcriptome data for pumpkin root tip) [49].

### Quantitative analysis by qRT-PCR

The qRT-PCR analysis was conducted following a previously established method [34]. Briefly, RNA was extracted from 0.2 g of samples ground in liquid nitrogen using the TransZol kit (TransGen, Beijing, China). cDNA was synthesized using the Hscript kit (Vazyme, Nanjing, China), and subsequently diluted to a concentration of 200 ng/μL with ddH_2_O. The qRT-PCR assays were carried out using the ABI6500 instrument (ABI, California, USA). The expression levels were calculated using the 2^−ΔΔct^ method [51]. The primer sequences used for quantitative PCR are provided in Table S2 (see online supplementary material). The internal reference gene used in this study was *Actin*.

### Luciferase complementary imaging

According to the methodology described by a previous study [52], cDNA fragments of *CmoDREB2A* and *CmoNAC1* were cloned into pCAMBIA-nLUC and pCAMBIA-cLUC vectors, respectively. The aforementioned constructs were subsequently introduced into *Agrobacterium* GV3101 for the purpose of *Nicotiana benthamiana* transformation. Subsequently, the infiltrated plants were cultivated within a controlled greenhouse environment, adhering to standard growth conditions, for a period ranging from 2 to 5 days. Subsequently, the lower surface of the leaves was sprayed with D-Luciferin potassium salt (Beyotime, Shanghai, China), and the fluorescence activity was visualized using the NightSHADE LB985 system (Berthold, Germany). The LUC/REN ratio was determined using a dual luciferase reporter assay kit (Beyotime, Shanghai, China) and measured with the Tecan Infinite M200 Pro microplate reader (Tecan).

### GST Pull-down

Following the method previously described [52], the GST-fusion vector pGEX-4 T-2 and His-fusion vector pET28a were used to construct CmoNAC1-GST and CmoDREB2A-His, respectively. The resulting constructs were then introduced into *Escherichia coli* BL21 cells. Protein expression were subsequently induced overnight at 16°C with 0.2 mM isopropyl β-D-1-thiogalactopyranoside (IPTG). The protein was purified separately using GST SefinoseTM Resin and His-tag Purification Resin (Beyotime, Shanghai, China). For the purification of GST and CmoNAC1-GST, prepared glutathione Sepharose beads were rotated with 0.5 mg of purified protein for 2 hours at 4°C. Following this, the beads were washed four times. After removing the supernatant, 1-mL purified CmoDREB2A-His recombinant protein was incubated with the beads for 2 h and subsequently washed four times with PBS buffer before being eluted using reduced glutathione. Finally, the eluents were analyzed via western blotting.

### BiFC

The BiFC assay was performed as described previously [9]. The NE173 vector (containing a N-terminal of YFP) was used to construct the full-length CmoNAC1 with a stop codon, while the CE155 vector (containing a C-terminal of YFP) was employed for constructing the full-length CmoDREB2A without a stop codon. Two plasmids, CmoDREB2A-cYFP and nYFP-CmoNAC1, were co-injected into tobacco leaves using Agrobacterium GV3101. Subsequently, these constructs were introduced into *Nicotiana benthamiana* by transformation using Agrobacterium GV3101. The transformed tobacco plants were grown under normal greenhouse conditions for 2–3 days before observing the red, green, and yellow fluorescence signals using laser scanning confocal microscopy.

### Subcellular localization of CmoNAC1 and CmoDREB2A

According to the previous method [53], the CDS of *CmoNAC1* and *CmoDREB2A* were modified by removing the stop codons, followed by fusion into the 1305.4-GFP and 101-YFP vectors, resulting in the generation of CmoNAC1-GFP and CmoDREB2A-YFP recombinant plasmids. *Agrobacterium* GV3101 cells were transformed with the recombinant plasmids, and a mixture of *Agrobacterium* GV3101 cells containing the nuclear label protein (mCherry) was combined with the transformed bacterial solution. This combined solution was then used to transform tobacco leaves for immediate expression. The transformed tobacco plants were cultivated under normal greenhouse conditions for 2–3 days, after which laser scanning confocal microscopy was utilized to observe the red, green, and yellow fluorescence signals.

### Analysis of transcriptional activation activity of CmoDREB2A

Following the previously described protocol [53], Y2HGold monoclonal strains were transformed with PGBKT7-CmoDREB2A, along with both negative control (pGBKT7 empty vector) and positive control (pGADKT7–53, or pHis 53). After inoculation into SD-Trp liquid medium, the cultures were incubated overnight at 28°C with shaking at 200 rpm. The OD600 of the yeast solution was carefully adjusted to a value of 0.1. Following this adjustment, 10 μL of the diluted solution was separately spotted onto an SD/Trp-His-Ade plate. The plates were subsequently air-dried and placed in a 28°C incubator for 3–5 days to allow yeast growth and observation of results.

### Yeast one-hybrid

In accordance with a previous study [34], yeast cells were co-transformed with pGADKT7 vectors containing cDNA sequences of transcription factors and pHis vectors containing the binding site sequence repeated in series three times. A negative control was established by co-transforming yeast with an empty pGADKT7 vector and a pHis vector containing the repeated binding site sequence. The positive control involved yeast co-transformed with pHis 53 and PGADKT7–53. Subsequent to the cultivation of monoclonal strains on solid SD/−Leu-Trp medium, inoculation into SD/−Leu-Trp liquid medium ensued, with overnight incubation at 28°C on a shaking table set at 200 rpm, until reaching an OD600 of 0.8. The yeast solution was then meticulously adjusted to achieve a uniform OD600 value of 0.1. Further dilutions were made with sterilized ddH_2_O to concentrations of 10^−1^, 10^−2^, and 10^−3^ of the original concentration. These three diluted solutions were plated onto solid media, including SD/−Leu/−Trp, SD/−Trp-Leu-His +0 mM 3-AT, SD/−Trp-Leu-His +40 mM 3-AT, and SD/−Trp-Leu-His +80 mM 3-AT, followed by air-drying. The results were observed after 3 to 5 days of growth in a 28°C incubator. The cloning primers can be found in Table S1 (see online supplementary material).

### GUS histochemical staining and luciferase analysis

In line with the procedures described by a previous study [54], GUS histochemical staining and luciferase (LUC) was employed to detect the interaction between transcription factors and promoters. The binding site repeated three times in series was used as promoter sequence. A set of primers was designed to amplify the entire transcription factor sequence, and the resulting amplified fragment was incorporated into the pGreenII62-SK vector at the BamHI and XhoI restriction sites via homologous recombination. Similarly, the promoter’s binding sites were introduced into the pCAMBIA1301 vector (contains the GUS tag without 35S promoter) at the HindIII and NcoI sites and pGreenII0800-LUC vector at the KpnI and SmaI sites using homologous recombination. Subsequently, the constructed plasmids were transformed into competent *Agrobacterium* GV3101 cells for tobacco transformation. For GUS histochemical staining, after 3 days of tobacco infection, the tobacco leaves were stained at 37°C for 24 h with the strong staining GUS dyeing solution of Beijing O’BioLab Technology Co., Ltd, and then decolorized with 75% ethanol (v/v) at 80°C for 1 h, the blue color was observed and photographed. For LUC experiment, we used a dual luciferase reporter assay kit (Beyotime, Shanghai, China) and a Tecan Infinite M200 Pro (Tecan) to determine the ratio of LUC to renilla luciferase (REN). Fluorescence activity was observed using NightSHADE LB985 (Berthold, Germany).

### Electrophoretic mobility shift assay

As per the method outlined by a previous study [55], we used CmoNAC1-GST and CmoDREB2A-His proteins in the EMSA experiment, which were previously purified via Pull-down. The 6-FAM labeled probe was synthesized by Qingke Biology Co., Ltd (Beijing, China). To denature the two complementary probes, they were mixed in a 1:1 volume ratio, heated to 95°C for 3 minutes, and slowly cooled down to room temperature. This process was carried out in a PCR apparatus, denatured at 95°C for 2 minutes, and lowered to 75°C for 30 cycles, after which it was lowered to 1°C and 15°C for 1 minute every 30 seconds. The probe was then bound to the recombinant protein. Next, the combined sample was immersed in Tris-borate EDTA buffer and run on a gel for 60 minutes at 100 V and 4°C. A thorough rinse with deionized water was performed on the surface before swiftly relocating the offset plate to a dimly lit area for capturing images using a multicolor fluorescent chemiluminescence imager (FM 1038, Minnesota, USA, Bio-Techne Corporation).

### Pumpkin root transformation

Salt-tolerant ‘Fenglejinjia’ pumpkin seeds (*Cucurbita maxima* × *Cucurbita moschata*) were obtained from Hefei Fengle Seed Co., Ltd. Salt-sensitive ‘Jinchun No.4’ cucumber seeds (*Cucumis sativus* L.) were obtained from Tianjin Kerun Agricultural Technology Co., Ltd. These varieties serve as grafted cucumber materials for creating root knockout or overexpressed *CmoDREB2A*. The method proposed by Geng *et al.* [56] was employed to construct knockout (BsaI digestion) and overexpression (XabI and SacI digestion) vectors of *CmoDREB2A* (*CmoCh03G003350*) using pKSE403. *Agrobacterium* K599 (Weidi, Shanghai) was transformed with the pKSE403 vector (as a control), pKSE403 containing sgRNA targeting *CmoDREB2A*, and pKSE403 integrating *CmoDREB2A* CDS in accordance with the K599 specification. The transformed *Agrobacterium* K599 was then used to infect the pumpkin, which was subsequently transplanted and grafted by top insertion method. During the first 3 days post-grafting, it is recommended to maintain a humidity level of 100% and avoid exposing the grafted seedlings to light. Subsequently, gradual ventilation and light supplementation should be introduced. Once the grafted cucumber seedlings become viable, it is advisable to remove their non-red fluorescent root every 10 days. PCR amplification was performed on the DNA extracted from DsRed root samples, and Hi-TOM sequencing was conducted on the PCR products to evaluate gene editing efficiency. After root identification, grafted cucumber plants with root knockout or overexpressed *CmoDREB2A* were obtained. Next, the seedlings were transferred to a hydroponic cup with the following specifications: a mouth diameter of 90 mm, bottom diameter of 57 mm, and a height of 135 mm. The cup was filled with 400 mL of 1/2 modified Hoagland nutritional solution comprising of 1 mM MgSO_4_·7H_2_O, 4 mM CaCl_2_, 10 mM KNO_3_, 0.5 mM Ca(H_2_PO4)_2_·H_2_O, and 74.93 mg/L Coolaber trace element solids (DZPM0059-500G). The pH of the solution was adjusted to 6.8 using 1 mM KOH. The growth conditions included a temperature range of 28 ± 1°C during the day and 18 ± 1°C at night, a light intensity of 250 μmol m^−2^ s^−1^, a day/night cycle of 14/10 hours, and a humidity range of 70–75%. For the grafting of cucumber seedlings at the three-leaf and one-heart stage, a control group was established without the addition of NaCl to the nutrient solution. In contrast, a salt treatment group was set up with the addition of 75 mM NaCl to the nutrient solution. Three replicates were performed with six plants per replicate. Table S1 (see online supplementary material) contains a list of the primers used.

### Determination of phenotype and photosynthetic index

After being treated with 75 mM NaCl for 7 days, the morphological and photosynthetic parameters of cucumber plants on pumpkin rootstock with knockout or overexpression of *CmoDREB2A* were assessed. Leaf area, total root surface area, root volume, total root length, dry weight, SPAD value, *Fv*/*Fm*, net photosynthetic rate (Pn), intercellular CO_2_ concentration (Ci), and stomatal conductance (Gs) were determined according to previously established methods [34].

### Malondialdehyde (MDA) content and relative conductivity (REC)

After being treated with 75 mM NaCl for 7 days, we assessed the MDA content and REC of grafted cucumber plants with either knocked-out or overexpressed *CmoDREB2A* roots. For MDA and REC analysis, fresh leaf and root samples, each weighing 0.1 g, were utilized. The MDA content was measured in accordance with the methodology outlined in a prior publication [57]. REC calculations were based on an established method [58].

### Determination of K^+^ and Na^+^ content

After a 7-day exposure to salinity (75 mM NaCl), Na^+^ and K^+^ contents in grafted cucumber plants with either knocked-out or overexpressed *CmoDREB2A* roots were quantified in accordance with the methodology outlined in a prior publication [59]. Dry leaf and root samples underwent grinding into a fine powder, from which precisely 0.1 g of the powder was measured and transferred into a digestion tube. Subsequently, 5 mL of 98% H_2_SO_4_ was introduced into the tube, which was then subjected to digestion at 300°C for 30 minutes. Gradual addition of 30% H_2_O_2_ continued until the digestion solution became clear. After appropriate dilution, the concentration was determined using atomic absorption spectrophotometry (Varian Spectra AA220, California, USA).

### Determination of H_2_O_2_ and ABA content

H_2_O_2_ and ABA levels were determined in both the leaves and roots of the grafted cucumber plants treated with 75 mM NaCl for 3 hours. For H_2_O_2_ content determination, we employed the H_2_O_2_ Content Detection Kit supplied by the Nanjing Institute of Biotechnology Engineering [60]. Analysis of ABA content was conducted by Meiji Bioscience (Shanghai, China) Co., Ltd. The quantitative measurement of ABA concentration was carried out using LC-ESI-MS/MS (UHPLC-Qtrap).

### Transcriptome assay

Following a 3-hour treatment with 75 mM NaCl, the leaves and roots of grafted cucumber plants were rapidly frozen in liquid nitrogen and subsequently ground into a fine powder. The resulting samples were then sent to Meiji Bioscience (Shanghai, China) Co., Ltd. for transcriptome sequencing. The sequencing data was aligned using the Chinese Long cucumber genome V3 (http://cucurbitgenomics.org/organism/20) and *C. moschata* (Rifu) genome V1 (http://cucurbitgenomics.org/organism/9). Differential gene expression analysis was conducted using DESeq2 software [61], and gene expression levels were measured in TPM (transcripts per million). The criteria for differentially expressed gene included Log2(fold change value) >0.5 and P value <0.05. The raw data of transcriptome has been uploaded to the NCBI website (PRJNA1027268). Gene accession numbers of cucumber and pumpkin are listed in Table S3 (see online supplementary material).

### Data analysis and image processing

The statistical plots were generated using Rstudio 4.03. To assess the significance of differences, a one-way ANOVA was conducted, followed by Duncan’s multiple-range test (*P* < 0.05).

## Acknowledgements

This research was supported by grants from the National Natural Science Foundation of China (32372794, 31772357, 32072653), Natural Science Foundation of Hubei Province (2019CFA017), Ningbo Scientific and Technological Project (2021Z006), and the Fundamental Research Funds for the Central Universities (2662023YLPY008).

## Author contributions

Z.B. and L.Y. conceived and designed the experiments. Y.P., L.C., Y.W., L.W., S.G., H.C., and G.C. performed the experiments and analysed the data. Y.P. and Z.B. wrote the paper, and all authors read the final manuscript.

## Data availability

The authors declare that all the data necessary to support the study’s conclusions are included in the paper and the supplemental materials, or can be obtained upon request from the corresponding author.

## Conflict of interest statement

The authors declare that they have no competing interests.

## Supplementary data

Supplementary data is available at *Horticulture Research* online.

## Supplementary Material

Web_Material_uhae057
