# Supplementary material for: Pumpkin CmoDREB2A enhances salt tolerance of grafted cucumber through interaction with CmoNAC1 to regulate H2O2 and ABA signaling and K+/Na+ homeostasis
Source: Hortic Res. 2024 Feb 28;11(5):uhae057. doi: 10.1093/hr/uhae057 (PMC11077054; doi:10.1093/hr/uhae057)
Supplement: Web_Material_uhae057 [file web_material_uhae057.zip › Supplementary File for Review .pptx]

## Slide 1
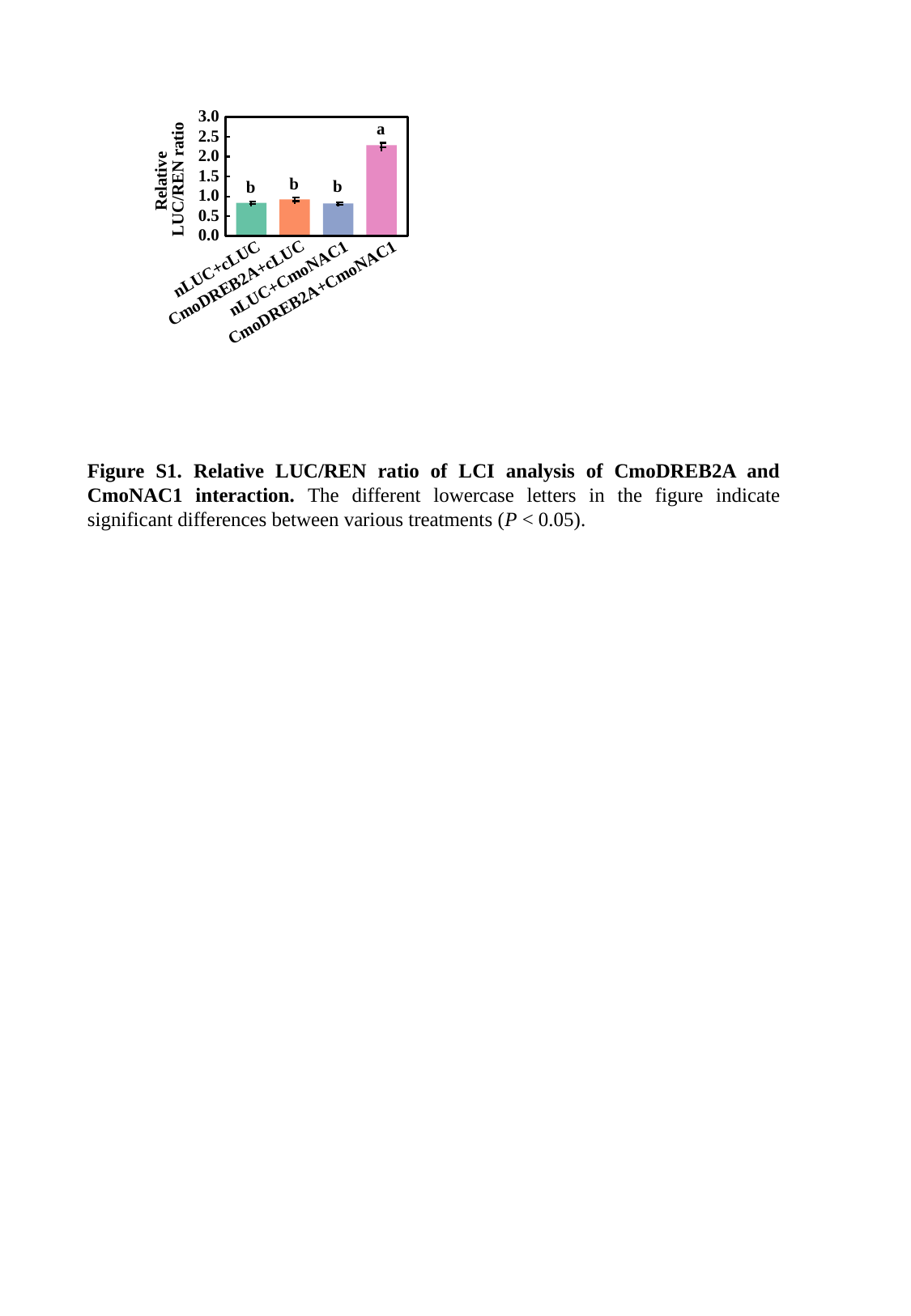

3.0
a
2.5
2.0
b
b
b
1.5
1.0
0.5
0.0
Relative
 LUC/REN ratio
nLUC+cLUC
nLUC+CmoNAC1
CmoDREB2A+cLUC
CmoDREB2A+CmoNAC1
Figure S1. Relative LUC/REN ratio of LCI analysis of CmoDREB2A and CmoNAC1 interaction. The different lowercase letters in the figure indicate significant differences between various treatments (P < 0.05).

## Slide 2
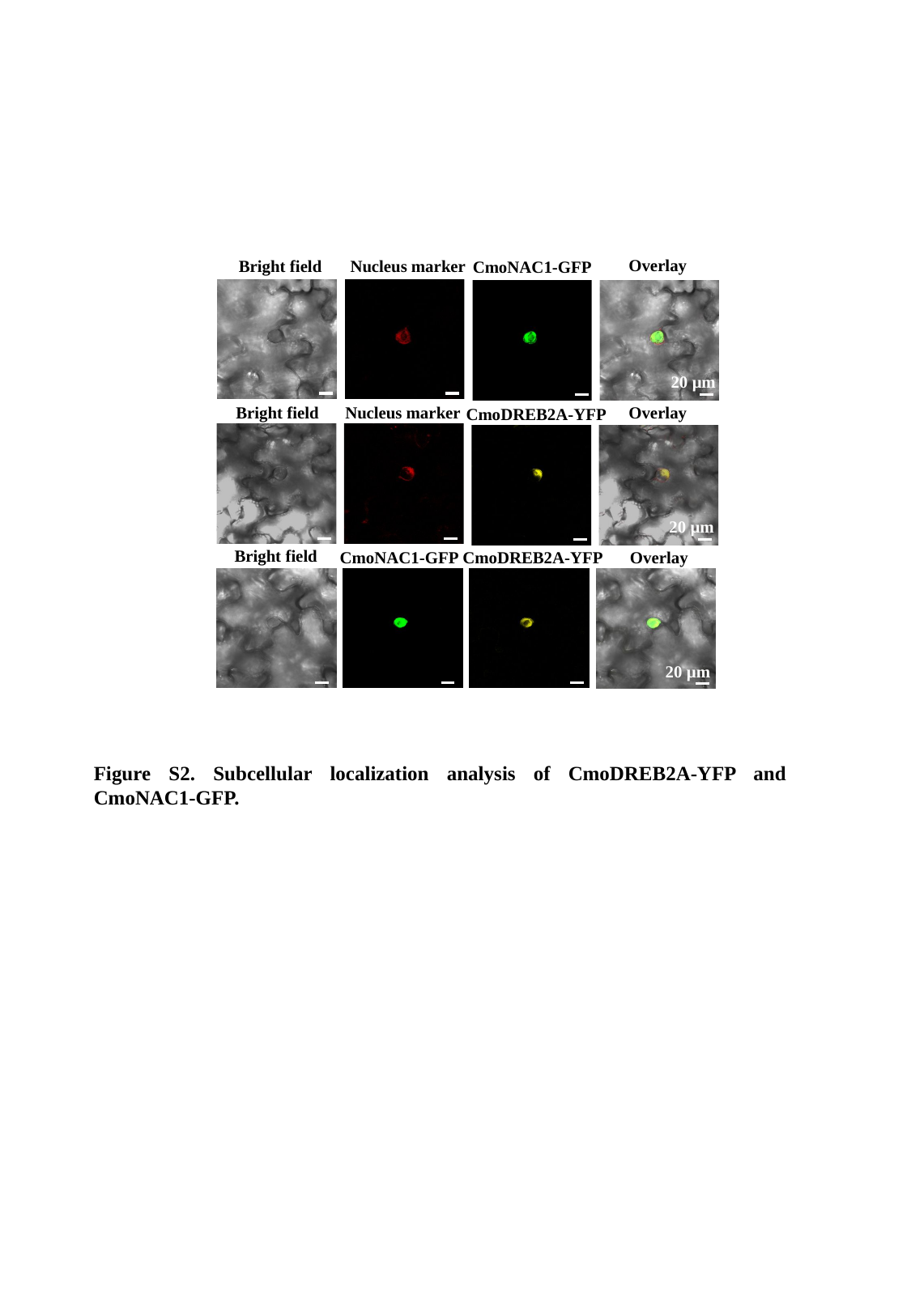

Overlay
Nucleus marker
Bright field
CmoNAC1-GFP
Nucleus marker
Overlay
Bright field
CmoDREB2A-YFP
Bright field
CmoDREB2A-YFP
Overlay
CmoNAC1-GFP
20 μm
20 μm
20 μm
Figure S2. Subcellular localization analysis of CmoDREB2A-YFP and CmoNAC1-GFP.

## Slide 3
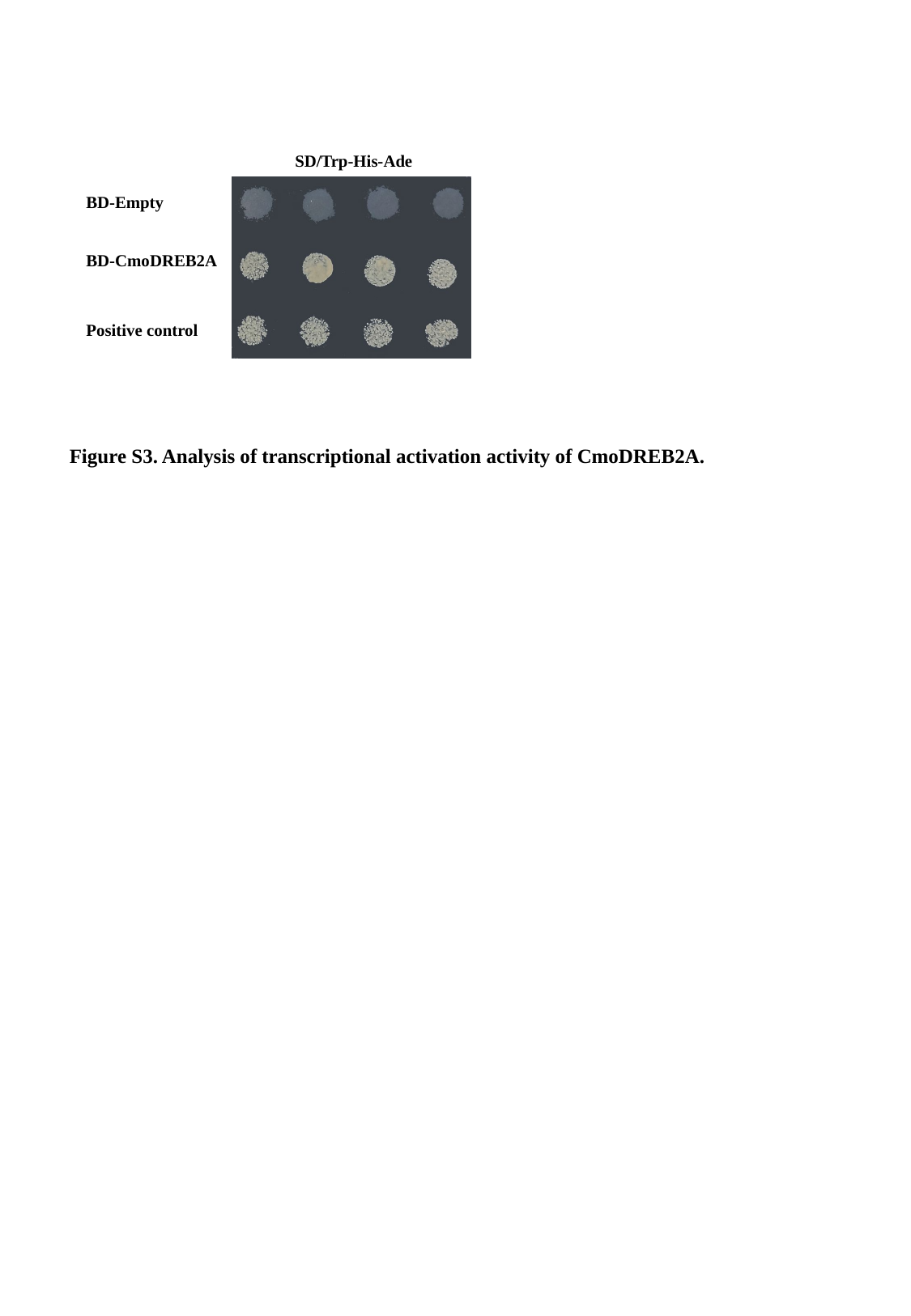

SD/Trp-His-Ade
BD-Empty
BD-CmoDREB2A
Positive control
Figure S3. Analysis of transcriptional activation activity of CmoDREB2A.

## Slide 4
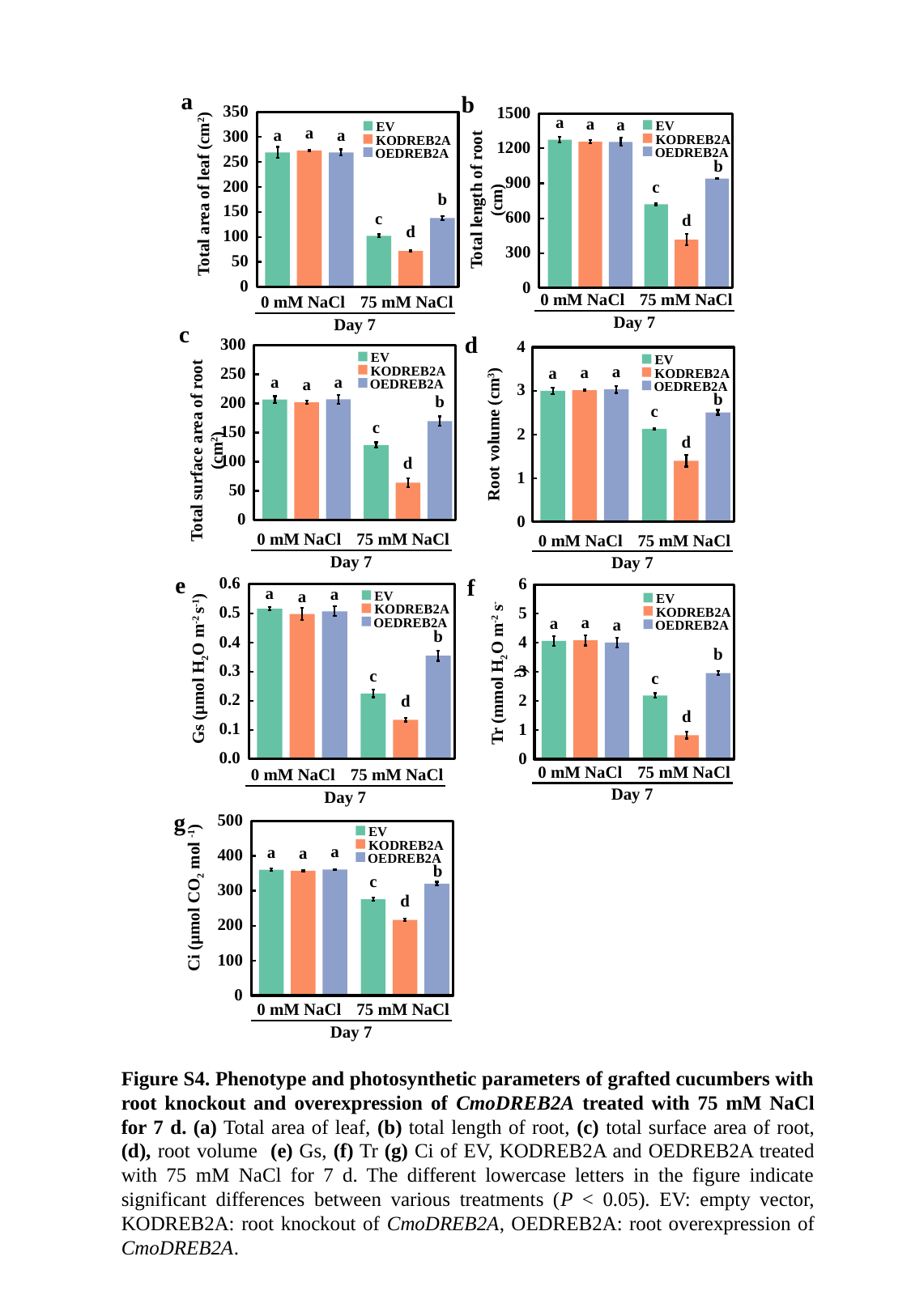

a
b
Total area of leaf (cm2)
350
EV
KODREB2A
 OEDREB2A
a
300
a
a
250
200
b
150
c
d
100
50
0
0 mM NaCl
75 mM NaCl
Day 7
Total length of root (cm)
1500
a
EV
KODREB2A
 OEDREB2A
a
a
1200
b
900
c
600
d
300
0
0 mM NaCl
75 mM NaCl
Day 7
c
d
Total surface area of root (cm2)
300
EV
KODREB2A
 OEDREB2A
250
a
a
a
b
200
c
150
100
d
50
0
0 mM NaCl
75 mM NaCl
Day 7
4
a
a
a
3
b
c
2
d
1
0
EV
KODREB2A
 OEDREB2A
Root volume (cm3)
0 mM NaCl
75 mM NaCl
Day 7
Gs (μmol H2O m-2 s-1)
0.6
a
EV
KODREB2A
 OEDREB2A
a
a
0.5
b
0.4
0.3
c
0.2
d
0.1
0.0
0 mM NaCl
75 mM NaCl
Day 7
e
f
6
5
a
a
a
4
b
3
c
2
d
1
0
Tr (mmol H2O m-2 s-1)
EV
KODREB2A
 OEDREB2A
0 mM NaCl
75 mM NaCl
Day 7
Ci (μmol CO2 mol -1)
500
EV
KODREB2A
 OEDREB2A
a
a
a
400
b
c
300
d
200
100
0
0 mM NaCl
75 mM NaCl
Day 7
g
Figure S4. Phenotype and photosynthetic parameters of grafted cucumbers with root knockout and overexpression of CmoDREB2A treated with 75 mM NaCl for 7 d. (a) Total area of leaf, (b) total length of root, (c) total surface area of root, (d), root volume (e) Gs, (f) Tr (g) Ci of EV, KODREB2A and OEDREB2A treated with 75 mM NaCl for 7 d. The different lowercase letters in the figure indicate significant differences between various treatments (P < 0.05). EV: empty vector, KODREB2A: root knockout of CmoDREB2A, OEDREB2A: root overexpression of CmoDREB2A.

## Slide 5
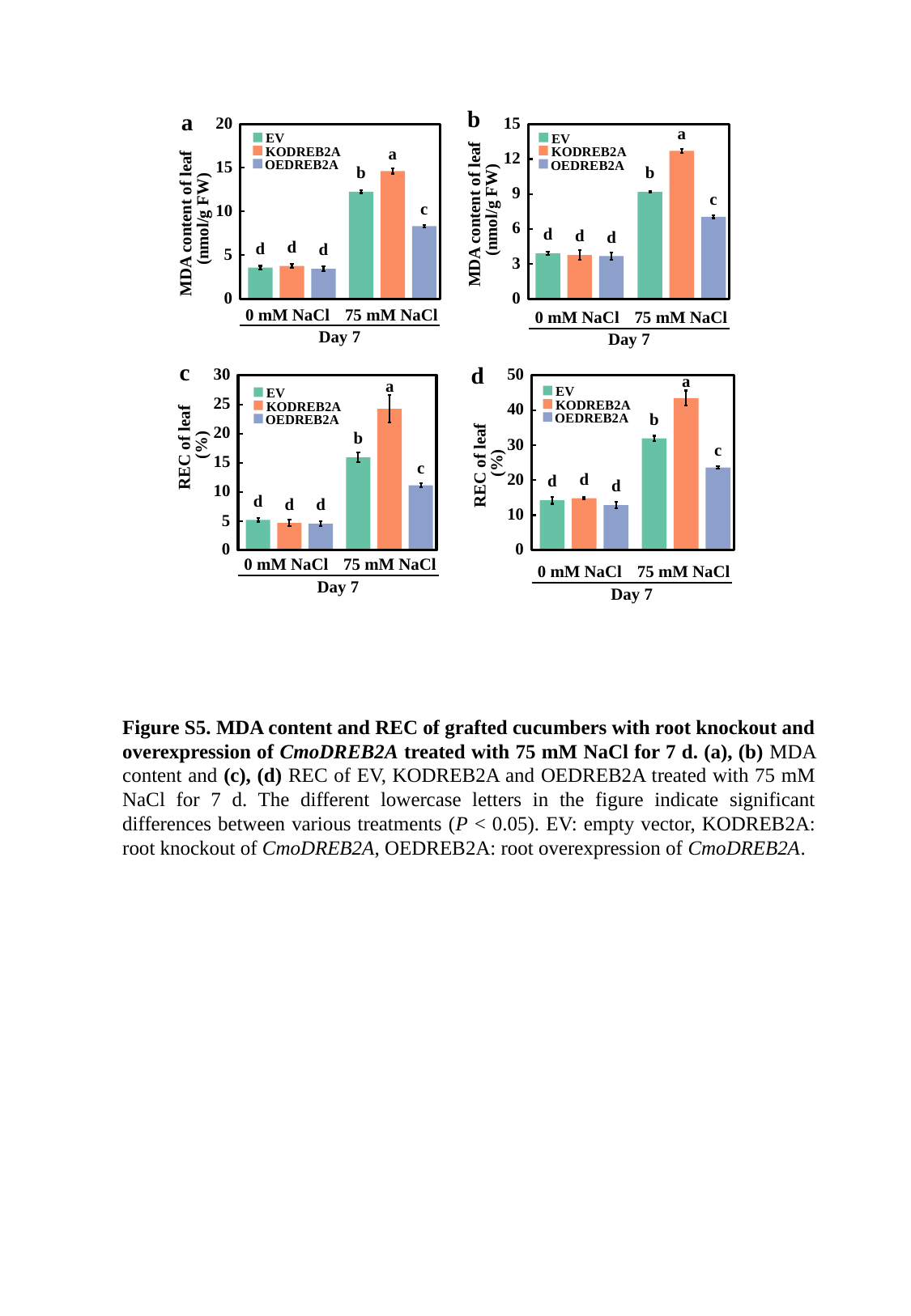

b
a
20
a
15
b
c
10
d
d
d
5
0
EV
KODREB2A
 OEDREB2A
MDA content of leaf
 (nmol/g FW)
0 mM NaCl
75 mM NaCl
Day 7
15
a
12
b
9
c
6
d
d
d
3
0
EV
KODREB2A
 OEDREB2A
MDA content of leaf
 (nmol/g FW)
0 mM NaCl
75 mM NaCl
Day 7
c
d
30
a
25
20
b
15
c
10
d
d
d
5
0
EV
KODREB2A
 OEDREB2A
REC of leaf
 (%)
0 mM NaCl
75 mM NaCl
Day 7
50
a
40
b
30
c
d
20
d
d
10
0
EV
KODREB2A
 OEDREB2A
REC of leaf
 (%)
0 mM NaCl
75 mM NaCl
Day 7
Figure S5. MDA content and REC of grafted cucumbers with root knockout and overexpression of CmoDREB2A treated with 75 mM NaCl for 7 d. (a), (b) MDA content and (c), (d) REC of EV, KODREB2A and OEDREB2A treated with 75 mM NaCl for 7 d. The different lowercase letters in the figure indicate significant differences between various treatments (P < 0.05). EV: empty vector, KODREB2A: root knockout of CmoDREB2A, OEDREB2A: root overexpression of CmoDREB2A.

## Slide 6
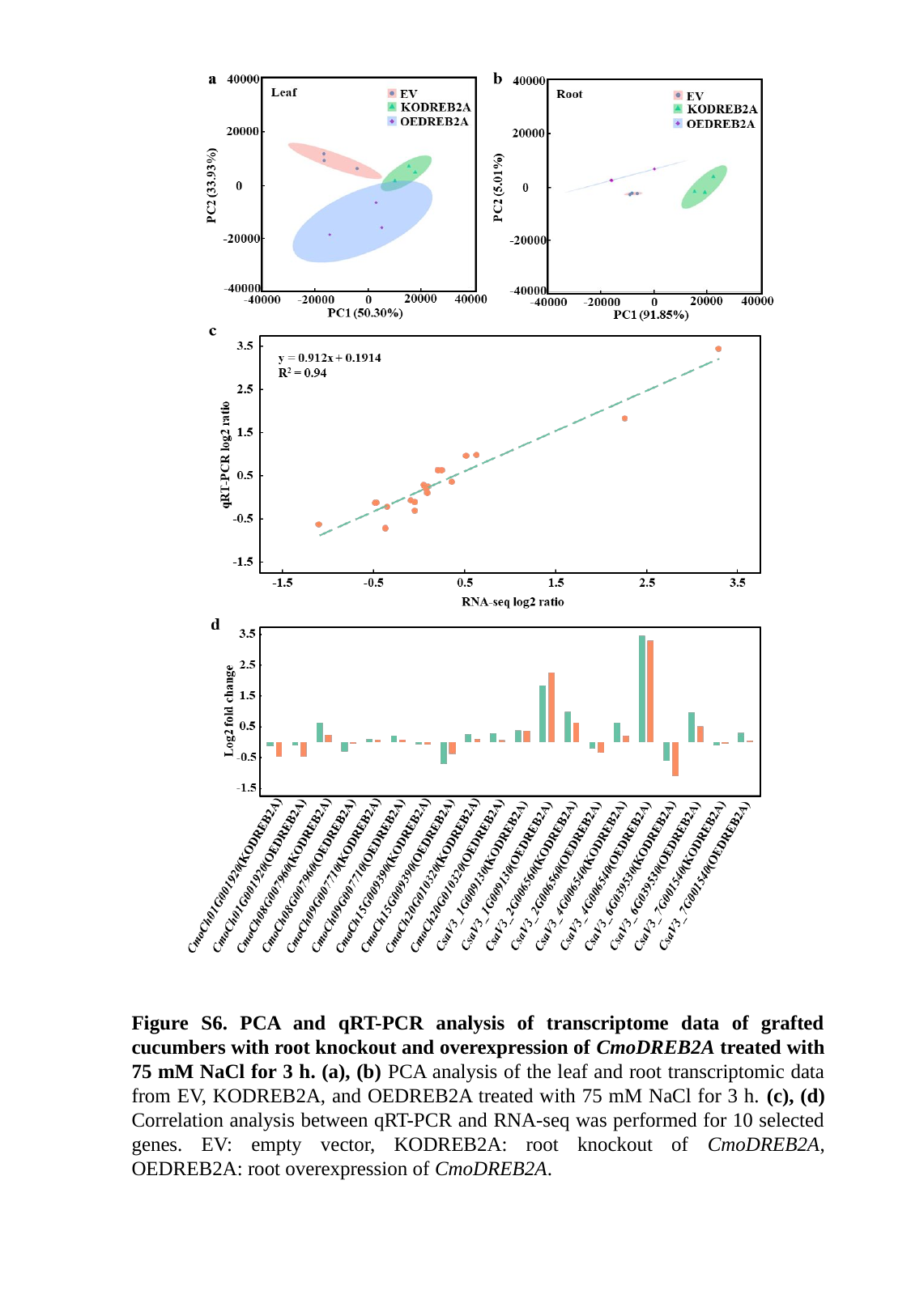

Figure S6. PCA and qRT-PCR analysis of transcriptome data of grafted cucumbers with root knockout and overexpression of CmoDREB2A treated with 75 mM NaCl for 3 h. (a), (b) PCA analysis of the leaf and root transcriptomic data from EV, KODREB2A, and OEDREB2A treated with 75 mM NaCl for 3 h. (c), (d) Correlation analysis between qRT-PCR and RNA-seq was performed for 10 selected genes. EV: empty vector, KODREB2A: root knockout of CmoDREB2A, OEDREB2A: root overexpression of CmoDREB2A.

## Slide 7
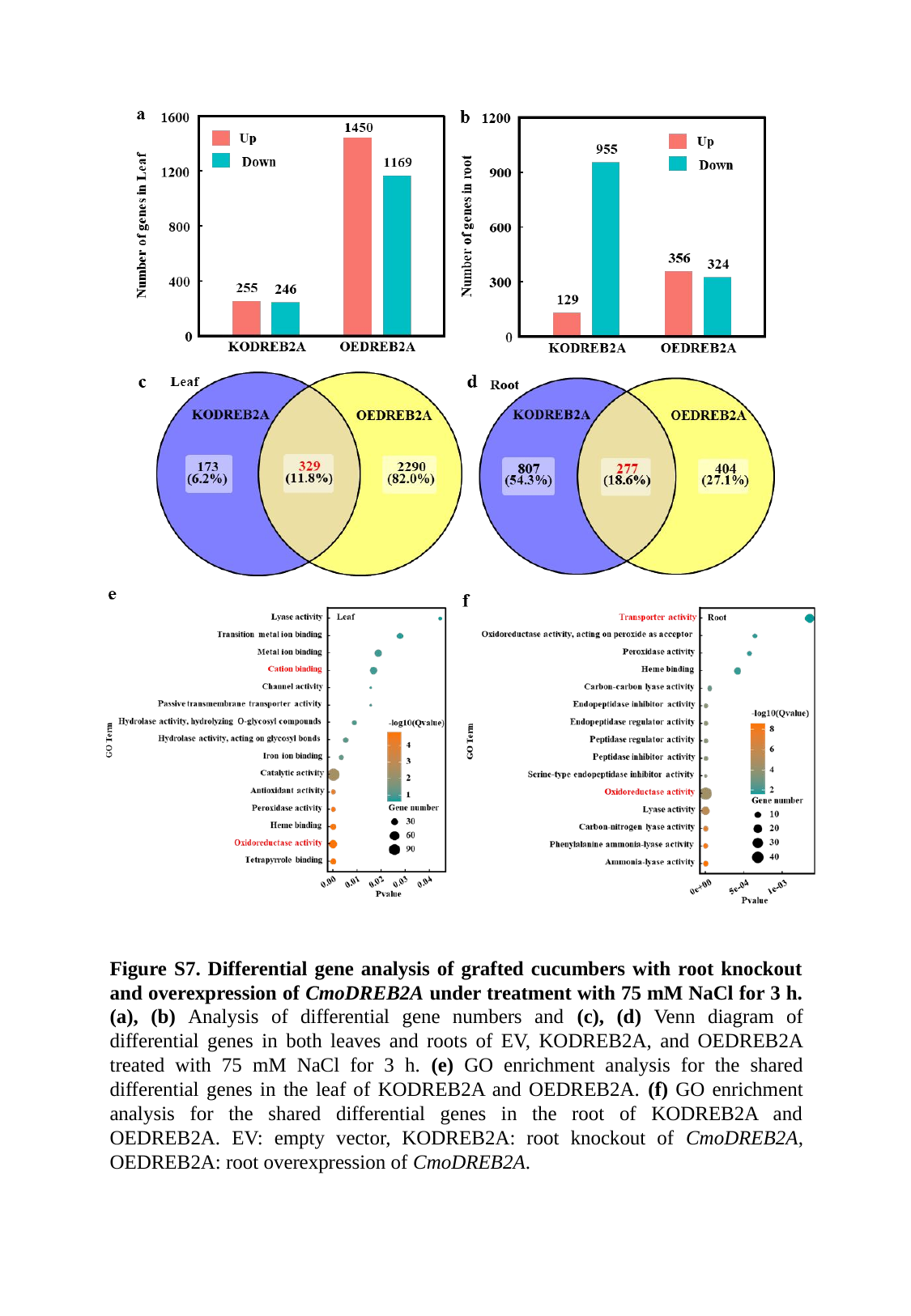

Figure S7. Differential gene analysis of grafted cucumbers with root knockout and overexpression of CmoDREB2A under treatment with 75 mM NaCl for 3 h. (a), (b) Analysis of differential gene numbers and (c), (d) Venn diagram of differential genes in both leaves and roots of EV, KODREB2A, and OEDREB2A treated with 75 mM NaCl for 3 h. (e) GO enrichment analysis for the shared differential genes in the leaf of KODREB2A and OEDREB2A. (f) GO enrichment analysis for the shared differential genes in the root of KODREB2A and OEDREB2A. EV: empty vector, KODREB2A: root knockout of CmoDREB2A, OEDREB2A: root overexpression of CmoDREB2A.

## Slide 8
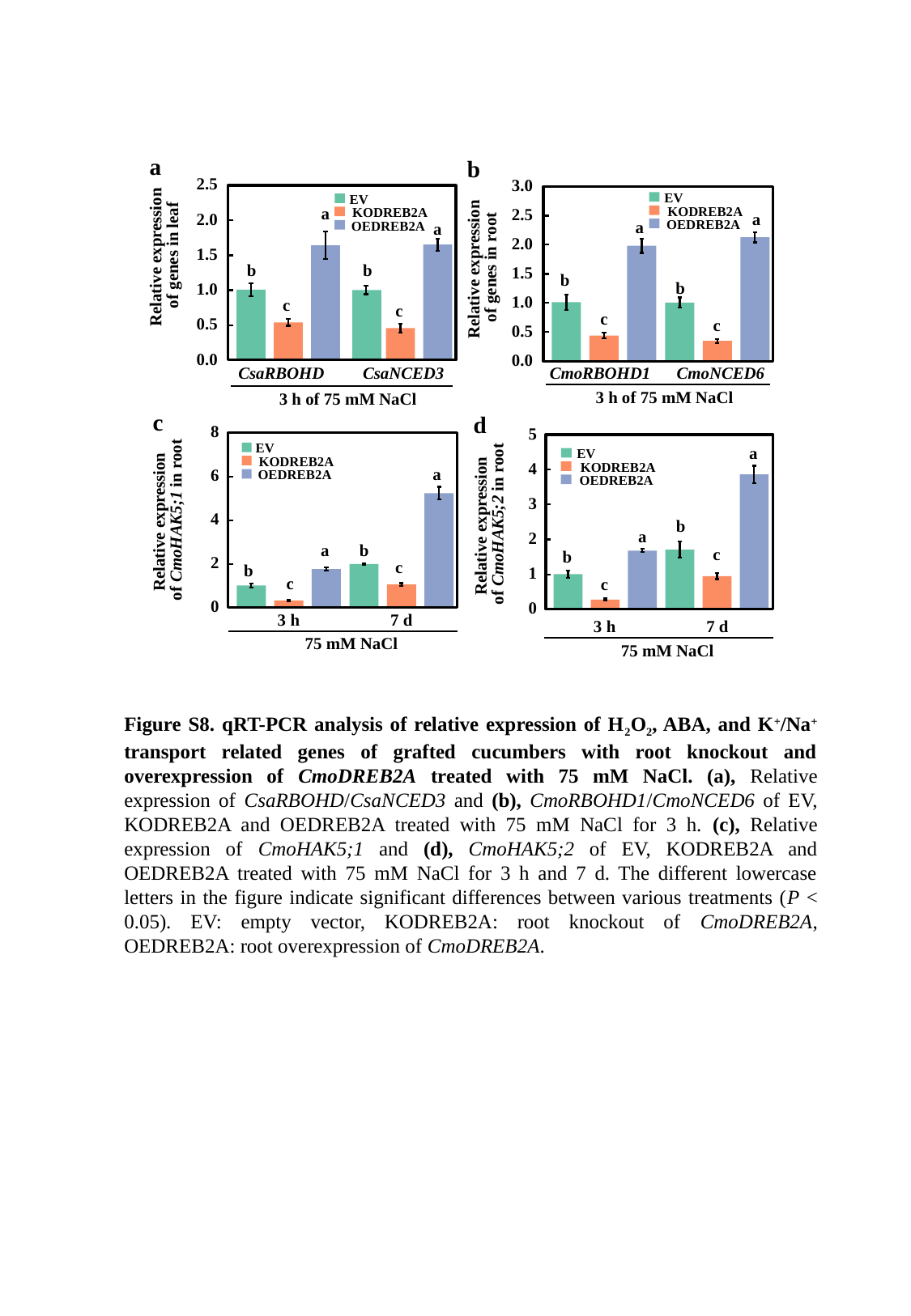

a
b
2.5
EV
KODREB2A
 OEDREB2A
a
2.0
a
Relative expression
of genes in leaf
1.5
b
b
1.0
c
c
0.5
0.0
CsaRBOHD
CsaNCED3
3 h of 75 mM NaCl
3.0
EV
KODREB2A
 OEDREB2A
2.5
a
a
2.0
Relative expression
of genes in root
1.5
b
b
1.0
c
c
0.5
0.0
CmoRBOHD1
CmoNCED6
3 h of 75 mM NaCl
c
d
8
6
4
2
0
EV
KODREB2A
 OEDREB2A
a
Relative expression
of CmoHAK5;1 in root
b
a
c
b
c
7 d
3 h
 75 mM NaCl
5
4
3
2
1
0
a
EV
KODREB2A
 OEDREB2A
Relative expression
of CmoHAK5;2 in root
b
a
c
b
c
7 d
3 h
 75 mM NaCl
Figure S8. qRT-PCR analysis of relative expression of H2O2, ABA, and K+/Na+ transport related genes of grafted cucumbers with root knockout and overexpression of CmoDREB2A treated with 75 mM NaCl. (a), Relative expression of CsaRBOHD/CsaNCED3 and (b), CmoRBOHD1/CmoNCED6 of EV, KODREB2A and OEDREB2A treated with 75 mM NaCl for 3 h. (c), Relative expression of CmoHAK5;1 and (d), CmoHAK5;2 of EV, KODREB2A and OEDREB2A treated with 75 mM NaCl for 3 h and 7 d. The different lowercase letters in the figure indicate significant differences between various treatments (P < 0.05). EV: empty vector, KODREB2A: root knockout of CmoDREB2A, OEDREB2A: root overexpression of CmoDREB2A.

## Slide 9
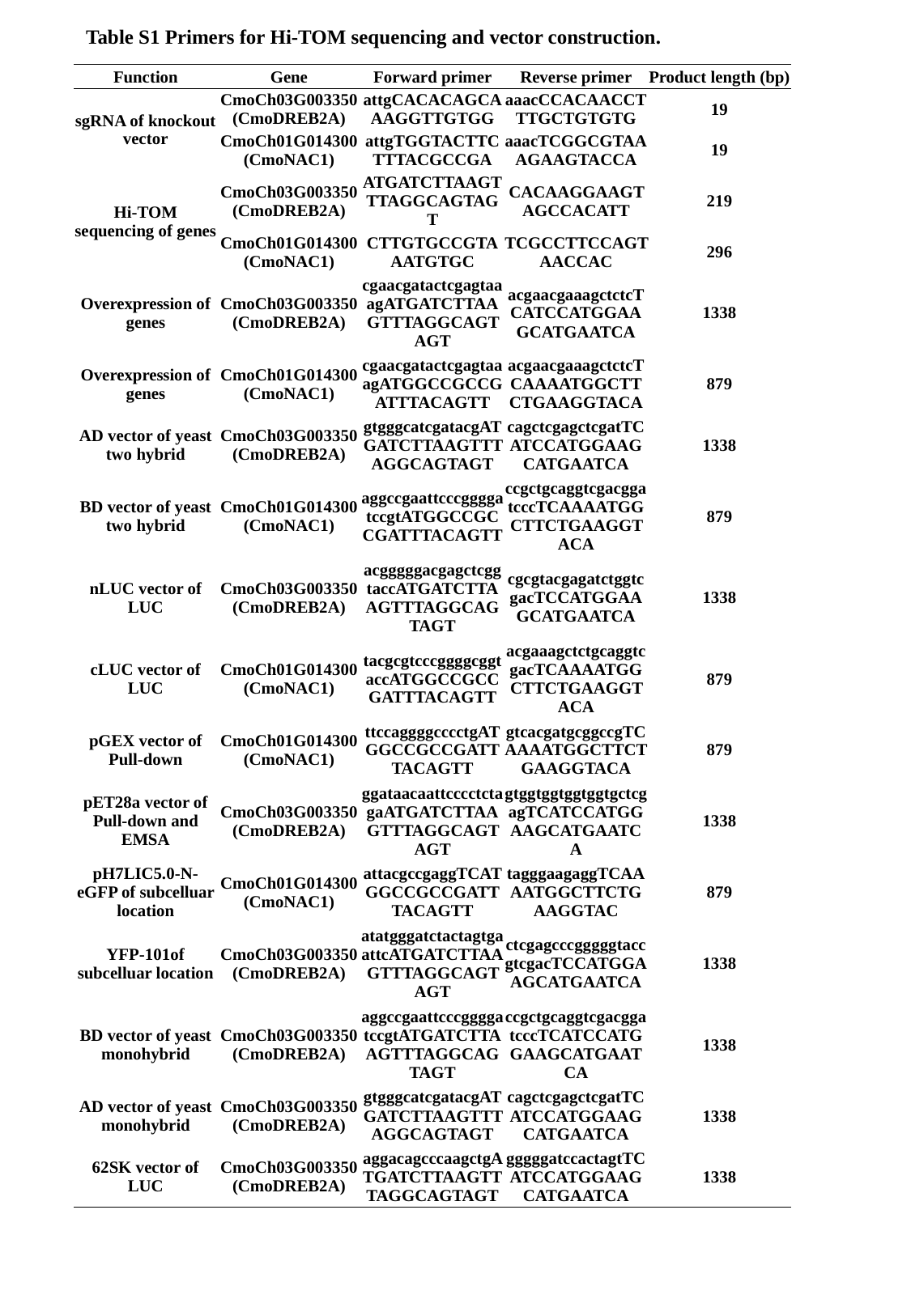

Table S1 Primers for Hi-TOM sequencing and vector construction.
| Function | Gene | Forward primer | Reverse primer | Product length (bp) |
| --- | --- | --- | --- | --- |
| sgRNA of knockout vector | CmoCh03G003350(CmoDREB2A) | attgCACACAGCAAAGGTTGTGG | aaacCCACAACCTTTGCTGTGTG | 19 |
| | CmoCh01G014300(CmoNAC1) | attgTGGTACTTCTTTACGCCGA | aaacTCGGCGTAAAGAAGTACCA | 19 |
| Hi-TOM sequencing of genes | CmoCh03G003350(CmoDREB2A) | ATGATCTTAAGTTTAGGCAGTAGT | CACAAGGAAGTAGCCACATT | 219 |
| | CmoCh01G014300(CmoNAC1) | CTTGTGCCGTAAATGTGC | TCGCCTTCCAGTAACCAC | 296 |
| Overexpression of genes | CmoCh03G003350(CmoDREB2A) | cgaacgatactcgagtaaagATGATCTTAAGTTTAGGCAGTAGT | acgaacgaaagctctcTCATCCATGGAAGCATGAATCA | 1338 |
| Overexpression of genes | CmoCh01G014300(CmoNAC1) | cgaacgatactcgagtaaagATGGCCGCCGATTTACAGTT | acgaacgaaagctctcTCAAAATGGCTTCTGAAGGTACA | 879 |
| AD vector of yeast two hybrid | CmoCh03G003350(CmoDREB2A) | gtgggcatcgatacgATGATCTTAAGTTTAGGCAGTAGT | cagctcgagctcgatTCATCCATGGAAGCATGAATCA | 1338 |
| BD vector of yeast two hybrid | CmoCh01G014300(CmoNAC1) | aggccgaattcccggggatccgtATGGCCGCCGATTTACAGTT | ccgctgcaggtcgacggatcccTCAAAATGGCTTCTGAAGGTACA | 879 |
| nLUC vector of LUC | CmoCh03G003350(CmoDREB2A) | acgggggacgagctcggtaccATGATCTTAAGTTTAGGCAGTAGT | cgcgtacgagatctggtcgacTCCATGGAAGCATGAATCA | 1338 |
| cLUC vector of LUC | CmoCh01G014300(CmoNAC1) | tacgcgtcccggggcggtaccATGGCCGCCGATTTACAGTT | acgaaagctctgcaggtcgacTCAAAATGGCTTCTGAAGGTACA | 879 |
| pGEX vector of Pull-down | CmoCh01G014300(CmoNAC1) | ttccaggggcccctgATGGCCGCCGATTTACAGTT | gtcacgatgcggccgTCAAAATGGCTTCTGAAGGTACA | 879 |
| pET28a vector of Pull-down and EMSA | CmoCh03G003350(CmoDREB2A) | ggataacaattcccctctagaATGATCTTAAGTTTAGGCAGTAGT | gtggtggtggtggtgctcgagTCATCCATGGAAGCATGAATCA | 1338 |
| pH7LIC5.0-N-eGFP of subcelluar location | CmoCh01G014300(CmoNAC1) | attacgccgaggTCATGGCCGCCGATTTACAGTT | tagggaagaggTCAAAATGGCTTCTGAAGGTAC | 879 |
| YFP-101of subcelluar location | CmoCh03G003350(CmoDREB2A) | atatgggatctactagtgaattcATGATCTTAAGTTTAGGCAGTAGT | ctcgagcccgggggtaccgtcgacTCCATGGAAGCATGAATCA | 1338 |
| BD vector of yeast monohybrid | CmoCh03G003350(CmoDREB2A) | aggccgaattcccggggatccgtATGATCTTAAGTTTAGGCAGTAGT | ccgctgcaggtcgacggatcccTCATCCATGGAAGCATGAATCA | 1338 |
| AD vector of yeast monohybrid | CmoCh03G003350(CmoDREB2A) | gtgggcatcgatacgATGATCTTAAGTTTAGGCAGTAGT | cagctcgagctcgatTCATCCATGGAAGCATGAATCA | 1338 |
| 62SK vector of LUC | CmoCh03G003350(CmoDREB2A) | aggacagcccaagctgATGATCTTAAGTTTAGGCAGTAGT | gggggatccactagtTCATCCATGGAAGCATGAATCA | 1338 |

## Slide 10
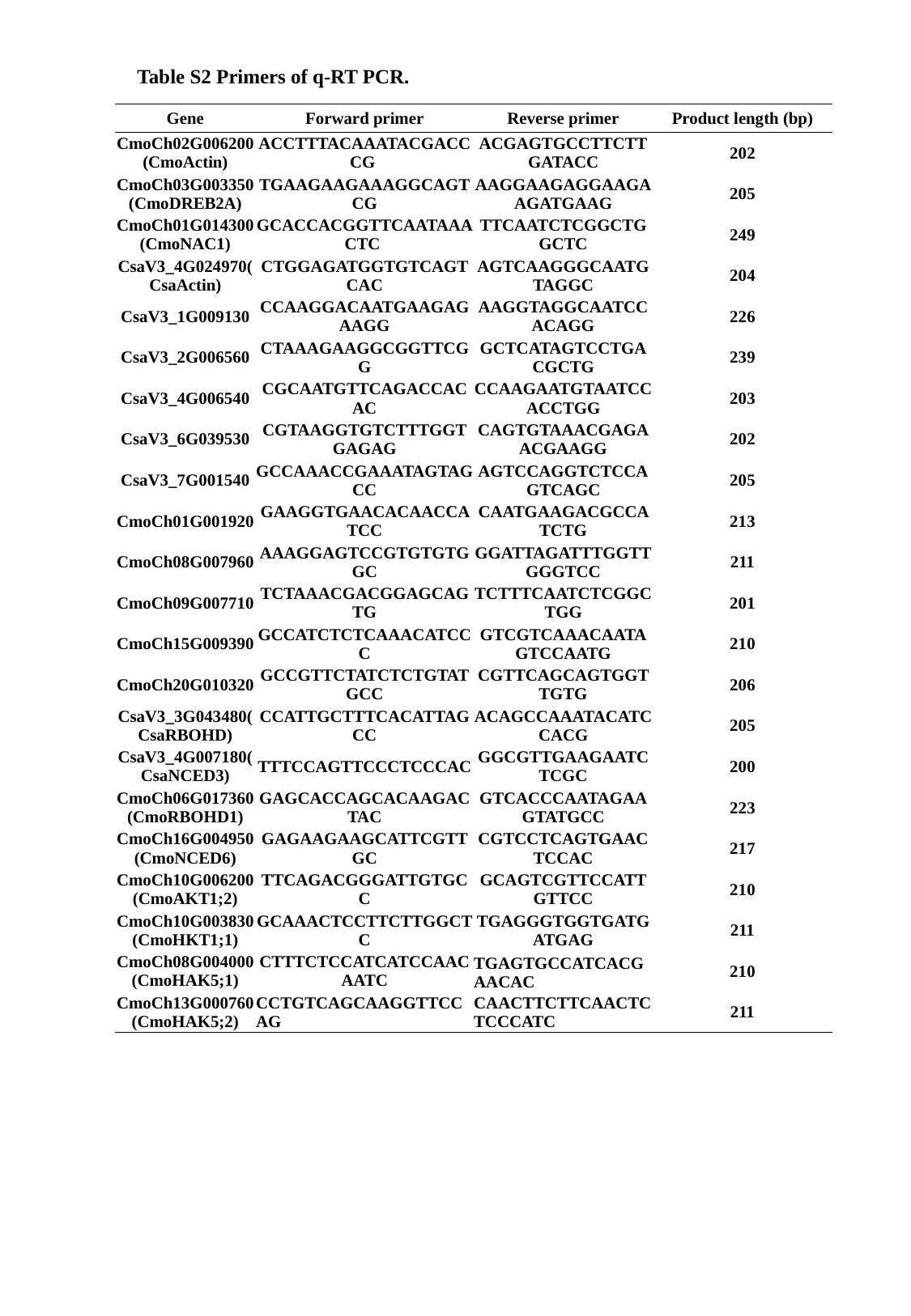

Table S2 Primers of q-RT PCR.
| Gene | Forward primer | Reverse primer | Product length (bp) |
| --- | --- | --- | --- |
| CmoCh02G006200(CmoActin) | ACCTTTACAAATACGACCCG | ACGAGTGCCTTCTTGATACC | 202 |
| CmoCh03G003350(CmoDREB2A) | TGAAGAAGAAAGGCAGTCG | AAGGAAGAGGAAGAAGATGAAG | 205 |
| CmoCh01G014300(CmoNAC1) | GCACCACGGTTCAATAAACTC | TTCAATCTCGGCTGGCTC | 249 |
| CsaV3\_4G024970(CsaActin) | CTGGAGATGGTGTCAGTCAC | AGTCAAGGGCAATGTAGGC | 204 |
| CsaV3\_1G009130 | CCAAGGACAATGAAGAGAAGG | AAGGTAGGCAATCCACAGG | 226 |
| CsaV3\_2G006560 | CTAAAGAAGGCGGTTCGG | GCTCATAGTCCTGACGCTG | 239 |
| CsaV3\_4G006540 | CGCAATGTTCAGACCACAC | CCAAGAATGTAATCCACCTGG | 203 |
| CsaV3\_6G039530 | CGTAAGGTGTCTTTGGTGAGAG | CAGTGTAAACGAGAACGAAGG | 202 |
| CsaV3\_7G001540 | GCCAAACCGAAATAGTAGCC | AGTCCAGGTCTCCAGTCAGC | 205 |
| CmoCh01G001920 | GAAGGTGAACACAACCATCC | CAATGAAGACGCCATCTG | 213 |
| CmoCh08G007960 | AAAGGAGTCCGTGTGTGGC | GGATTAGATTTGGTTGGGTCC | 211 |
| CmoCh09G007710 | TCTAAACGACGGAGCAGTG | TCTTTCAATCTCGGCTGG | 201 |
| CmoCh15G009390 | GCCATCTCTCAAACATCCC | GTCGTCAAACAATAGTCCAATG | 210 |
| CmoCh20G010320 | GCCGTTCTATCTCTGTATGCC | CGTTCAGCAGTGGTTGTG | 206 |
| CsaV3\_3G043480(CsaRBOHD) | CCATTGCTTTCACATTAGCC | ACAGCCAAATACATCCACG | 205 |
| CsaV3\_4G007180(CsaNCED3) | TTTCCAGTTCCCTCCCAC | GGCGTTGAAGAATCTCGC | 200 |
| CmoCh06G017360(CmoRBOHD1) | GAGCACCAGCACAAGACTAC | GTCACCCAATAGAAGTATGCC | 223 |
| CmoCh16G004950(CmoNCED6) | GAGAAGAAGCATTCGTTGC | CGTCCTCAGTGAACTCCAC | 217 |
| CmoCh10G006200(CmoAKT1;2) | TTCAGACGGGATTGTGCC | GCAGTCGTTCCATTGTTCC | 210 |
| CmoCh10G003830(CmoHKT1;1) | GCAAACTCCTTCTTGGCTC | TGAGGGTGGTGATGATGAG | 211 |
| CmoCh08G004000(CmoHAK5;1) | CTTTCTCCATCATCCAACAATC | TGAGTGCCATCACGAACAC | 210 |
| CmoCh13G000760(CmoHAK5;2) | CCTGTCAGCAAGGTTCCAG | CAACTTCTTCAACTCTCCCATC | 211 |

## Slide 11
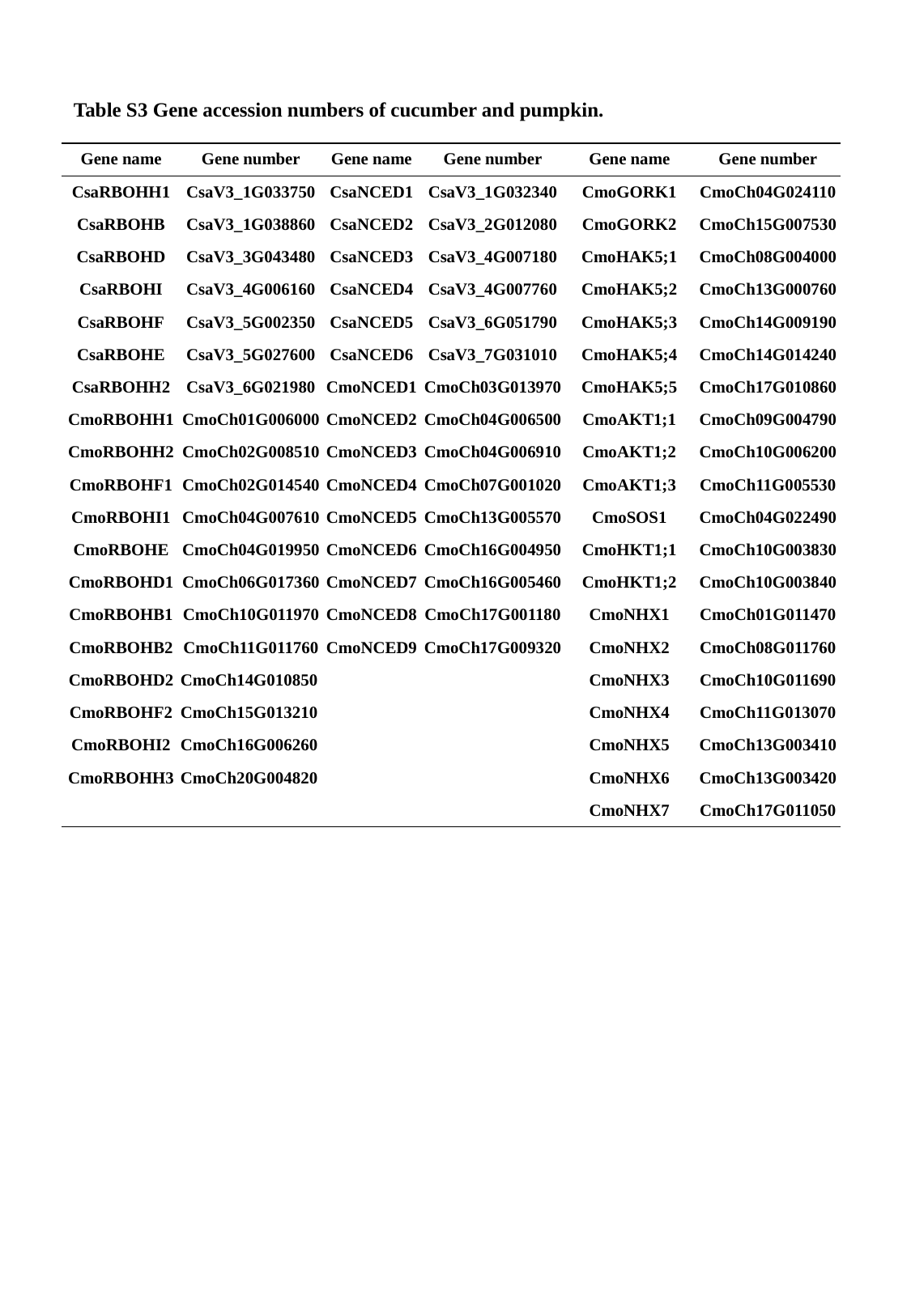

Table S3 Gene accession numbers of cucumber and pumpkin.
| Gene name | Gene number | Gene name | Gene number | Gene name | Gene number |
| --- | --- | --- | --- | --- | --- |
| CsaRBOHH1 | CsaV3\_1G033750 | CsaNCED1 | CsaV3\_1G032340 | CmoGORK1 | CmoCh04G024110 |
| CsaRBOHB | CsaV3\_1G038860 | CsaNCED2 | CsaV3\_2G012080 | CmoGORK2 | CmoCh15G007530 |
| CsaRBOHD | CsaV3\_3G043480 | CsaNCED3 | CsaV3\_4G007180 | CmoHAK5;1 | CmoCh08G004000 |
| CsaRBOHI | CsaV3\_4G006160 | CsaNCED4 | CsaV3\_4G007760 | CmoHAK5;2 | CmoCh13G000760 |
| CsaRBOHF | CsaV3\_5G002350 | CsaNCED5 | CsaV3\_6G051790 | CmoHAK5;3 | CmoCh14G009190 |
| CsaRBOHE | CsaV3\_5G027600 | CsaNCED6 | CsaV3\_7G031010 | CmoHAK5;4 | CmoCh14G014240 |
| CsaRBOHH2 | CsaV3\_6G021980 | CmoNCED1 | CmoCh03G013970 | CmoHAK5;5 | CmoCh17G010860 |
| CmoRBOHH1 | CmoCh01G006000 | CmoNCED2 | CmoCh04G006500 | CmoAKT1;1 | CmoCh09G004790 |
| CmoRBOHH2 | CmoCh02G008510 | CmoNCED3 | CmoCh04G006910 | CmoAKT1;2 | CmoCh10G006200 |
| CmoRBOHF1 | CmoCh02G014540 | CmoNCED4 | CmoCh07G001020 | CmoAKT1;3 | CmoCh11G005530 |
| CmoRBOHI1 | CmoCh04G007610 | CmoNCED5 | CmoCh13G005570 | CmoSOS1 | CmoCh04G022490 |
| CmoRBOHE | CmoCh04G019950 | CmoNCED6 | CmoCh16G004950 | CmoHKT1;1 | CmoCh10G003830 |
| CmoRBOHD1 | CmoCh06G017360 | CmoNCED7 | CmoCh16G005460 | CmoHKT1;2 | CmoCh10G003840 |
| CmoRBOHB1 | CmoCh10G011970 | CmoNCED8 | CmoCh17G001180 | CmoNHX1 | CmoCh01G011470 |
| CmoRBOHB2 | CmoCh11G011760 | CmoNCED9 | CmoCh17G009320 | CmoNHX2 | CmoCh08G011760 |
| CmoRBOHD2 | CmoCh14G010850 | | | CmoNHX3 | CmoCh10G011690 |
| CmoRBOHF2 | CmoCh15G013210 | | | CmoNHX4 | CmoCh11G013070 |
| CmoRBOHI2 | CmoCh16G006260 | | | CmoNHX5 | CmoCh13G003410 |
| CmoRBOHH3 | CmoCh20G004820 | | | CmoNHX6 | CmoCh13G003420 |
| | | | | CmoNHX7 | CmoCh17G011050 |
